# Axially Chiral Biphenyl Compound‐Based Thermally Activated Delayed Fluorescent Materials for High‐Performance Circularly Polarized Organic Light‐Emitting Diodes

**DOI:** 10.1002/advs.202000804

**Published:** 2020-06-14

**Authors:** Zhen‐Long Tu, Zhi‐Ping Yan, Xiao Liang, Lei Chen, Zheng‐Guang Wu, Yi Wang, You‐Xuan Zheng, Jing‐Lin Zuo, Yi Pan

**Affiliations:** ^1^ State Key Laboratory of Coordination Chemistry Collaborative Innovation Center of Advanced Microstructures Jiangsu Key Laboratory of Advanced Organic Materials School of Chemistry and Chemical Engineering Nanjing University Nanjing 210023 P. R. China

**Keywords:** axially chiral biphenyl compound, circularly polarized electroluminescence, circularly polarized luminescence, organic light‐emitting diodes, thermally activated delayed fluorescence

## Abstract

To boost intrinsic circularly polarized luminescence (CPL) properties of chiral emitters, an axially chiral biphenyl unit is inlaid in thermally activated delayed fluorescent (TADF) skeleton, urging the participation of chiral source in frontier molecular orbital distributions. A pair of enantiomers, (*R*)‐BPPOACZ and (*S*)‐BPPOACZ, containing the cyano as electron‐withdrawing moieties and carbazole and phenoxazine as electron‐donating units are synthesized and separated. The circularly polarized TADF enantiomers exhibit both high photoluminescence quantum yield of 86.10% and excellent CPL activities with maximum dissymmetry factor |*g*_PL_| values of almost 10^−2^ in solution and 1.8 × 10^−2^ in doped film, which are among the best values of previously reported small chiral organic materials. Moreover, the circularly polarized organic light‐emitting diodes based on the TADF enantiomers achieve the maximum external quantum efficiency of 16.6% with extremely low efficiency roll‐off. Obvious circularly polarized electroluminescence signals with |*g*_EL_| values of 4 × 10^−3^ are also recorded.

## Graphical abstract

Taking a new design strategy, a pair of axially chiral thermally activated delayed fluorescent enantiomers are developed with high photoluminescence quantum yield and excellent circularly polarized luminescence signals. Moreover, the resulting circularly polarized organic light‐emitting diodes achieve both high external quantum efficiency with low efficiency roll‐off and obvious circularly polarized electroluminescence signals.

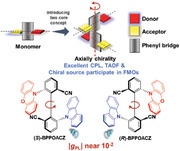

Circularly polarized (CP) light has recently drawn growing attention because of its potential applications in optical data storage, quantum computing, bioresponsive imaging, optical spintronics, and 3D display, etc.^[^ ^1^ ^]^ Comparing to CP light arising from filtered nonpolarized light, chiral luminogenic materials can enable CP light emission directly without brightness loss, which is particularly favorable for circularly polarized organic light‐emitting diodes (CP‐OLEDs) to develop efficient and cost‐effective 3D display and CP light sources.^[^ ^2^, ^3^ ^]^ Generally, the intensity of CP light is characterized by the dissymmetry factor (*g*) with the formula of *g* = 2 × Δ*I*/*I =* 2 × (*I*_L_ − *I*_R_)/(*I*_L_ + *I*_R_); where *I*_L_ and *I*_R_ represent the intensity of left‐CPL and right‐CPL.

Although there have been several researches on CP‐OLEDs by utilizing chiral luminogenic materials, it remains a challenge to pursue high device performances with both high *g* values and efficiencies simultaneously. In 1997, Meijer and co‐workers first detected the circularly polarized electroluminescence (CPEL) from a chiral polymer‐based CP‐OLEDs.^[^ ^4a^ ^]^ Since then, various CPL materials have been applied in CP‐OLEDs, including conjugated polymers^[^ ^4^ ^]^ and luminescent lanthanide complexes.^[^ ^5^ ^]^ Suffering from low brightness, insufficient efficiency, and serious efficiency roll‐off,^[^ ^4^, ^5^ ^]^ the above‐mentioned materials are too far apart for future 3D display and optoelectronic technology.

To overcome these defects in conventional CP‐OLEDs, the chiral TADF materials show unique advantages as emitters in OLEDs. TADF materials can harness both singlet and triplet excitons for light emission without noble metal^[^ ^6^ ^]^ by reverse intersystem crossing (RISC) process, benefiting from small energy gap (Δ*E*_ST_) between S_1_ and T_1_ levels.^[^ ^7^, ^8^ ^]^ Meanwhile, directly connecting chiral units with TADF skeletons can endow the achiral emitters chirality with CPL capability. In this context, the first reported CP‐TADF material possessed a chiral carbon sandwiching between donor (D) and acceptor (A), showing photoluminescence dissymmetry factors (|*g*_PL_|) of 1.1 × 10^−3^.^[^ ^2^ ^]^ In 2016, Pieters’ group pioneered binaphthol (BINOL)‐based CP‐TADF material by binding BINOL and CN–Cz‐based TADF molecule achieving |*g*_PL_| of 1.3 × 10^−3^.^[^ ^9a^ ^]^ Then, Chen group combined 1,2‐diaminocyclohexane with aromatic‐imide‐based TADF skeleton, developing a pair of enantiomers with |*g*_PL_| of 1.1 × 10^−3^. CP‐OLEDs based on the enantiomers achieved EQE_max_ of 19.8% and electroluminescence dissymmetry factors |*g*_EL_| as high as 2.3 × 10^−3^.^[^ ^9b,c^ ^]^ Later, Tang's group developed BINOL‐based CP‐TADF materials utilizing similar design strategy with |*g*_PL_| value in the order of 1 × 10^−3^. Surprisingly, the corresponding CP‐OLEDs exhibited |*g*_EL_| value of 0.02 with a twenty times of amplification and EQE_max_ of 9.3%.^[^ ^3^ ^]^ In 2019, our group reported CP‐OLEDs with a satisfying EQE_max_ of 32.6% and |*g*_EL_| value of 2 × 10^−3^ by using octahydro‐BINOL‐based CP‐TADF materials.^[^ ^9d^ ^]^ Very recently, Chen's group developed a new axially chiral CP‐TADF emitters with |*g*_EL_| value of 10^−2^.^[^ ^9e,f^ ^]^

In most cases, the CPEL performances of CP‐OLEDs maintain the same order with the intrinsic CPL properties of corresponding CP‐TADF materials. The inferior |*g*_PL_| values of reported CP‐TADF emitters really frustrate CPEL performances of CP‐OLEDs (Figure S2, Supporting Information). Therefore, it is crucial to develop novel CP‐TADF materials with intense CPL properties and high luminescent efficiencies. Fundamentally introducing novel molecule design strategies would bring an optional chance to break the bottleneck of practical CP‐TADF materials, instead of simply connecting chiral sources with TADF molecules.

In this paper, axially chiral biphenyl unit is inlaid in the TADF skeleton based on cyano–carbazole (CN–Cz) and CN–phenoxazine (POA) subunits for a pair of enantiomers, namely (*R*)‐BPPOACZ and (*S*)‐BPPOACZ, respectively (Figure S3, Supporting Information). Notably, the biphenyl unit not only is the cornerstone of introducing the chirality, but also acts as a bridge connecting the donors and acceptors in the TADF skeleton. On the one hand, two pairs of donors and acceptors on the sides of biphenyl unit can cause great steric hindrance, which generates torsion between two phenyl rings and prevents the racemization of the axially chiral biphenyl unit. On the other hand, the twist between biphenyl unit also stabilizes the D–A structures in two TADF cores, minimizing the Δ*E*_ST_ consequently. This overlap of functions makes biphenyl unit a chiral source to actively participate in the frontier molecular orbital (FMO) distributions, which subsequently breeds the excellent CPL properties with maximum |*g*_PL_| values of 9.7 × 10^−3^ in toluene and 1.8 × 10^−2^ in doped film. The enantiomers show small Δ*E*_ST_ values of 0.04 eV and high photoluminescence quantum yield (PLQY) of 86.10% in toluene under nitrogen atmosphere. The doped CP‐OLEDs based on the enantiomers also exhibited intense CPEL signals with a |*g*_EL_| of ≈ 4.0 × 10^−3^ with EQE_max_ up to 16.6% with extremely low efficiency roll‐off.

Racemic BPPOACZ was prepared by a three‐step process through Cu‐catalyzed classical Ullmann coupling reaction from 2‐bromo‐3‐fluorobenzonitrile followed by nucleophilic substitution with phenoxazine and carbazole, respectively (Scheme S1, Supporting Information). The optically pure enantiomers of (*R/S*)‐BPPOACZ were then separated adopting a chiral column with enantiomeric excesses (ee) >99% (Figure S4, Supporting Information). As shown in **Figure** **1**a, BPPOACZ is a stable axially chiral emitter with two CN‐based TADF units coupled in a sterically hindering configuration. Through further gradient sublimation (Table S1, Supporting Information), high‐quality single crystal was obtained and no racemization occurred benefiting from the large steric hindrance surrounding the axially bridge. Taking (*R*)‐BPPOACZ for example, it exhibits a highly twisted conformation confirmed by the single‐crystal X‐ray diffraction analysis (Figure 1b; Table S2, Supporting Information). Besides, the distance of 2.73 Å between donor on the side of biphenyl unit and acceptor on the other side enables C–H···*π* intramolecular interactions. United with the *π*···*π* interactions between two donor groups with cofacial alignment distance of 3.59 Å and dihedral angle <20° (Figure S5, Supporting Information), these strong intramolecular noncovalent interactions may suppress the nonradiative decay.^[^ ^8^ ^]^ Multiwfn software was used to calculate the functions of reduced density gradient (RDG) and Sign(*λ*_2_)*ρ* by single‐crystal structures. RDG analysis confirms the presence of obvious intramolecular attractive interactions (green region in Figure S5 in the Supporting Information) between two donor groups and CN groups.^[^ ^8e^ ^]^ In addition, the BPPOACZ material possesses good thermal stability with decomposition temperature (5% weight loss) of 338 °C (**Table** **1**; Figure S6, Supporting Information).

**Figure 1:**
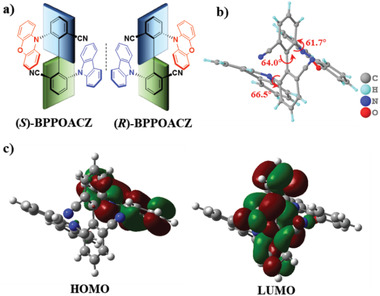
a) Molecular design and chemical structures of the TADF enantiomers (*S*)‐BPPOACZ and (*R*)‐BPPOACZ. b) Single crystal of (*R*)‐BPPOACZ. c) HOMO and LUMO distributions of (*R*)‐BPPOACZ.

**Table 1:** Summary of photophysical and electrochemical properties of (*rac*)‐BPPOACZ

Compound	*T*_d_ ^a)^ [°C]	*λ*_abs_ ^b)^ [nm]	*λ*_PL_ ^b)^ [nm]	*λ*_PL_ ^c)^ [nm]	*Ф*_PL_ ^d)^ [%]	*τ*_p_ ^e)^ [ns]	*τ*_d_ ^e)^ [µs]	HOMO/LUMO^f)^ [eV]	*E*_g_ ^g)^ [eV]	*E*_AE_(S_1_)^h)^ [eV]	*E*_AE_(T_1_)^h)^ [eV]	Δ*E*_ST_ ^i)^ [eV]
(*rac)*‐BPPOACZ	338	295, 320, 337, 380	384, 543	527	86.10	57	1.1	−5.35/−2.61	2.74	2.79	2.76	0.04

a) Decomposition temperature (5% weight loss), determined from thermal gravimetric analysis (Figure S6, Supporting Information);

b) Peaks of absorption and fluorescence spectra in toluene (5.0 × 10^−5^ m);

c) Peak of fluorescence spectrum in doped film ((*rac*)‐BPPOACZ:26DCzPPy = 2:10);

d) Absolute PLQY measured in toluene under nitrogen atmosphere (5.0 × 10^−5^ m);

e) Lifetime of the prompt component and delayed component determined from transient PL measurement in toluene under nitrogen atmosphere at 288 K;

f) HOMO calculated from the oxidation potential in 10^−4^ m acetonitrile solution by cyclic voltammetry with ferrocene as the internal standard with the formula of HOMO = −[*E*_ox_ − *E*_(Fc/Fc+)_ + 4.8] eV and LUMO calculated from HOMO − *E*_g_;

g) Estimated from the onset of the absorption spectrum with the formula of *E*_g_ = 1240/*λ*_onset_;

h) Adiabatic emission energies estimated from the onset of the fluorescence and phosphorescence spectra at 77 K in toluene (5.0 × 10^−5^ m);

i) Calculated from *E*_AE_(S_1_) − *E*_AE_(T_1_).

To gain a better insight into the geometrical structures and FMO distributions as well as the corresponding energy levels of (*R*)‐BPPOACZ, time‐dependent density‐functional theory (TD‐DFT) using B3LYP/6‐31G(d) method was performed. From the geometry optimization of (*R*)‐BPPOACZ, it was found that the HOMO is mainly located on the phenoxazine moiety (91.53%), and the LUMO is mainly located on the biphenyl rings (80.22%) and cyano units (14.75%) (Figure 1c), which illustrates obvious spatial separation (Table S3, Supporting Information). The distribution pattern also indicates clear involvement of the chiral biphenyl part which could potentially contribute to stronger electronic circular dichroism (ECD) and CPL activities. Furthermore, the compound also shows reversible redox curve in acetonitrile (Figure S7, Supporting Information), and its HOMO/LUMO energy levels were calculated as −5.35/−2.61 eV (Table 1), respectively.

The absorption spectrum of (*rac*)‐BPPOACZ in toluene displays normal n–π* and *π*–π* transition bands before 420 nm (**Figure** **2**a) and also shows a slight change as the solvent polarity increases (Figure S8a, Supporting Information). Due to two relatively independent D–*π*–A subunits, (*rac*)‐BPPOACZ in toluene exhibits two emissions peaking at 384 and 543 nm,^[^ ^8^, ^10^ ^]^ while the main emission presents a significant redshift with the increase of the solvent polarity: from 514 nm in *n*‐hexane to 582 nm in dichloromethane (Figure S8b, Supporting Information). But the secondary peak around 400 nm disappears in doped films with a widely used host of 26DczPPy (2,6‐bis[3‐(9*H*‐carbazol‐9‐yl)phenyl]pyridine) and doped devices, probably as a result of intramolecular charge transfer (ICT) and highly strengthened and dominant intermolecular CT process in condensed state.^[^ ^8c^ ^]^ The ICT characteristics can be verified from Lippert–Mataga plot in various solvents (Table S5 and Figure S9, Supporting Information). The Δ*E*_ST_ estimated from fluorescence and phosphorescence (77 K) spectra (Figure S10, Supporting Information) is only 0.04 eV, which could facilitate RISC process, leading to high radiative efficiency for achieving efficient TADF material. The absolute PLQY of (*rac*)‐BPPOACZ in degassed toluene solution (5 × 10^−5^ m) is 86.10%, determined by an integrating sphere (Figure S12, Supporting Information). As shown in Figure S11 (Supporting Information), the prompt emission of (*rac*)‐BPPOACZ exhibits fluorescence lifetime (*τ*_p_) of 57 ns and delayed fluorescence component with lifetime (*τ*_d_) of 1.1 µs in toluene under nitrogen atmosphere. The short TADF lifetimes may help in suppressing the triplet–triplet annihilation (TTA) processes in the devices for realizing low efficiency roll‐off.

**Figure 2:**
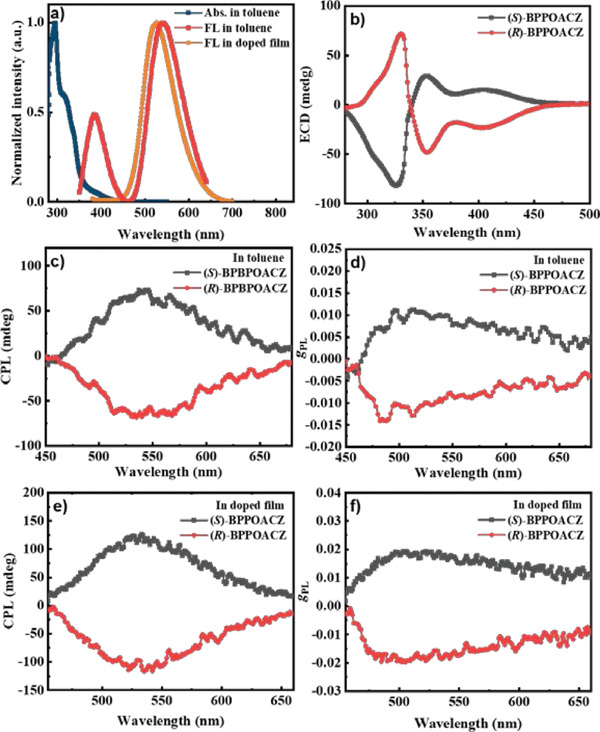
a) Absorption and fluorescence spectra of (*rac*)‐BPPOACZ in toluene (5.0 × 10^−5^ m), and fluorescence spectra of doped film ((*rac*)‐BPPOACZ:26DCzPPy = 2:10) at room temperature. b) ECD spectra of (*R/S*)‐BPPOACZ in toluene (5.0 × 10^−5^ m). c) CPL spectra of (*R/S*)‐BPPOACZ in toluene (5.0 × 10^−5^ m). d) *g*_PL_ versus wavelength curves of (*R/S*)‐BPPOACZ in toluene. e) CPL spectra in doped film ((*R/S*)‐BPPOACZ:26DCzPPy = 2:10). f) *g*_PL_ versus wavelength curves in doped film ((*R/S*)‐BPPOACZ:26DCzPPy = 2:10).

The chiroptical properties of the (*R/S*)‐BPPOACZ enantiomers in the ground and excited states were investigated by ECD and CPL spectra measurements (Figure 2b,c,e), respectively. As illustrated in Figure 2b, the ECD spectra of (*R*)‐BPPOACZ and (*S*)‐BPPOACZ display an obvious mirror–image relationship. The strong Cotton effects in short wavelength region around 300 nm could be assigned to the characteristic absorption of chiral biphenyl unit. Meanwhile, the Cotton effects with the maximum band around 350 and 400 nm are attributed to the absorption of the D–A electronic structure of TADF units. Subsequently, the almost mirror–image CPL spectra of the enantiomers were also observed (Figure 2c) with the *g*_PL_ of +9.7 × 10^−3^ for (*S*)‐BPPOACZ and −8.7 × 10^−3^ for (*R*)‐BPPOACZ in toluene solutions (Figure 2d), respectively. The *g*_PL_ values of (*R/S*)‐BPPOACZ in different solutions around the emission maxima in various solutions are quite similar (Figure S13, Supporting Information). The chiroptical properties of codeposited films ((*R/S*)‐BPPOACZ:26DCzPPy = 2:10) were significantly improved with *g*_PL_ values of +1.85 × 10^−2^ and −1.72 × 10^−2^ (Figure 2f), respectively. The high *g*_PL_ of 1.85 × 10^−2^ is the one of the highest values among all reported chiral organic materials.

Considering the high PLQY and CPL properties, (*rac*)‐BPPOACZ and (*R/S*)‐BPPOACZ enantiomers were employed as emitters for fabricating CP‐OLEDs. The devices of ITO/HATCN (hexaazaztriphenylene‐hexacabonitrile, 6.5 nm)/TAPC (di‐[4‐(*N*,*N*‐ditolylamino)phenyl]cyclohexane, 30 nm)/(*rac*)‐BPPOACZ, (*R*)‐BPPOACZ or (*S*)‐BPPOACZ (8 wt%):26DCzPPy (20 nm)/TmPyPB (1,3,5‐tri(*m*‐pyrid‐3‐yl‐phenyl)benzene, 70 nm)/LiF (1 nm)/Al (100 nm) were named as D‐RAC, D‐R and D‐S, respectively (Figure S14, Supporting Information).

Due to almost identical performances of the devices based on (*rac*)‐BPPOACZ, (*R*)‐BPPOACZ or (*S*)‐BPPOACZ, the D‐RAC was chosen as an example to investigate device properties in details. The EL spectrum of device D‐RAC based on (*rac*)‐BPPOACZ shows emission with peak of 537 nm and the CIE 1931 coordinates of (0.36, 0.57) (Figure S15, Supporting Information). From the luminance‐voltage curves in **Figure** **3**b (purple line) it can be observed that D‐RAC exhibits a maximum brightness of 35 711 cd m^−2^. It is noteworthy that the device D‐RAC displays high efficiencies with a maximum current efficiency (*η*_c,max_) of 56.9 cd A^−1^, a maximum external quantum efficiency (EQE_max_) of 16.6% and a maximum power efficiency (*η*_p,max_) of 36.4 lm W^−1^, respectively (Figure 3c,d and **Table** **2**). Furthermore, the device D‐RAC also exhibits very low efficiency roll‐offs, and the EQE can still maintain 15.2% at the brightness of 1000 cd m^−2^, and 14.9% even at the brightness of 5000 cd m^−2^, corresponding to 8.4%, and 10.2% decrease in EQE, respectively. The device performances are promising for CP‐OLEDs based on chiral organic emitters (Table 2).

**Figure 3:**
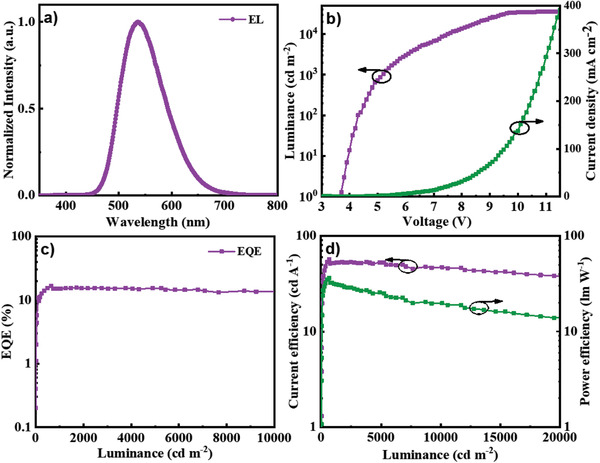
Device D‐RAC performances: a) EL spectrum, b) current density–luminance–voltage curves, c) EQE–luminance curves, and d) current‐efficiency/power‐efficiency–luminance curves.

**Table 2:** Summary of key data of OLED performances

Device	*V*_on_ ^a)^ [V]	*L*_max_ ^b)^ [cd m^−2^]	*η*_c,max_ ^c)^ [cd A^−1^]	EQE_max_ ^d)^ [%]	EQE_1000/5000/10 000_ ^e)^ [%]	*η*_p,max_ ^f)^ [lm W^−1^]	*λ*_EL_ ^g)^ [nm]	CIE^h)^ (*x*, *y*)
D‐RAC	3.7	35 711	56.9	16.6	15.2/14.9/13.8	36.4	537	(0.36, 0.57)
D‐S	3.8	38 128	61.0	17.8	15.2/14.3/12.6	42.6	537	(0.36, 0.57)
D‐R	3.7	37 899	51.9	15.1	14.8/13.4/12.2	31.5	537	(0.36, 0.57)

a) Turn‐on voltage;

b) Maximum luminance;

c) Maximum current efficiency;

d) Maximum external quantum efficiency;

e) External quantum efficiencies at 1000, 5000, and 10 000 cd m^−2^;

f) Maximum power efficiency;

g) Peak of electroluminescence;

h) CIE 1931 coordinate.

The superior efficiency and low efficiency roll‐off of D‐RAC can be attributed to the following reasons. First, C–H···*π* and *π*···*π* intramolecular interactions observed in single crystal collectively rigidify the molecular structures and facilitate the suppression of motional radiationless relaxation, enabling the efficient transformation of excitonic energy into photons. Second, the twisted conformation is favored to impede close packing and weaken intermolecular interactions. Thus, concentration‐caused TTA can be suppressed greatly.^[^ ^8^ ^]^ Furthermore, small Δ*E*_ST_ of 0.04 eV accelerates the RISC process and short DF lifetime of 1.1 µs reduces the exciton annihilation, thus the efficiency roll‐off induced by TTA is prevented.^[^ ^11^ ^]^ The experimental result are compared with a simulative efficiency roll‐off behavior by taking triplet–triplet annihilation TTA into account, which conforms to the discussion (Figure S16, Supporting Information).

Then the CPEL characteristics of devices D‐R and D‐S with the enantiomers were independently investigated and their chiroptical properties were measured at 5 V in JASCO CPL‐300 with practical adjustments (Figure S1, Supporting Information). As illustrated in **Figure** **4**, the D‐R and D‐S display obviously opposite CPEL signals and remain the same directions as the CPL signals of (*R*)‐BPPOACZ and (*S*)‐BPPOACZ materials, indicating that the axially chiral biphenyl unit is responsible for the observed CP effect in both photoluminescence and electroluminescence processes. At the emission maximum of 537 nm, the *g*_EL_ values reach +4.5 × 10^−3^ for (*S*)‐BPPOACZ‐based D‐S and −2.8 × 10^−3^ for (*R*)‐BPPOACZ‐based D‐R, respectively, which are relatively high compared with the most existing reports (Figure S2, Supporting Information).

**Figure 4:**
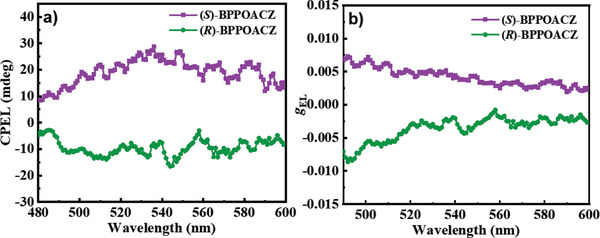
a) CPEL spectra of devices D‐R and D‐S. b) *g*_EL_ curves of devices D‐R and D‐S.

According to the hypothesis of Barnes and co‐workers,^[^ ^12^ ^]^ the lower |*g*_EL_| values of doped devices D‐R and D‐S would be caused by cancellation effects in ensemble measurements of a randomly oriented bulk sample. Moreover, as reported by Di Bari and co‐workers, the spiral direction of CP light would reverse after the reflection from cathode, which would impair the degree of circular polarization.^[^ ^5^, ^6^ ^]^ However, the strong intrinsic CPL property of (*R/S*)‐BPPOACZ may spark more creative design strategy and theoretical efforts in CP‐TADF materials and CP‐OLEDs.

In conclusion, a pair of axially chiral biphenyl compound based TADF enantiomers of (*R*)‐BPPOACZ and (*S*)‐BPPOACZ were synthesized and investigated. The enantiomers represent excellent TADF properties with a small Δ*E*_ST_ value of 0.04 eV, a short DF lifetime of 1.1 µs, and a high PLQY of 86.10%. Moreover, axially chiral biphenyl unit forces the chiral sources to take part in FMOs distributions, which may contribute to the significant CPL properties with |*g*_PL_| values up to 9.7 × 10^−3^ in toluene solutions and 1.85 × 10^−2^ in doped films. The device with (*rac*)‐BPPOACZ shows good performances with a brightness over 30 000 cd m^−2^, an EQE up to 16.6% with low efficiency roll‐offs. Furthermore, the CP‐OLEDs based on (*R/S*)‐BPPOACZ enantiomers display the symmetrical CPEL signals with the *g*_EL_ factors of up to +4.5 × 10^−3^. Our strategy provides a feasible pathway toward design of CP‐TADF materials with excellent CPL and luminous properties for efficient CP‐OLEDs.

[CCDC of 1962429 contain the supplementary crystallographic data for this paper. These data can be obtained free of charge from The Cambridge Crystallographic Data Centre via www.ccdc.cam.ac.uk/data_request/cif.]

## Conflict of Interest

The authors declare no conflict of interest.

## Supporting information

Supporting Information
