# Supplementary material for: Axially Chiral Biphenyl Compound‐Based Thermally Activated Delayed Fluorescent Materials for High‐Performance Circularly Polarized Organic Light‐Emitting Diodes
Source: Adv Sci (Weinh). 2020 Jun 14;7(15):2000804. doi: 10.1002/advs.202000804 (PMC7404162; doi:10.1002/advs.202000804)
Supplement: Supplementary file 1 — Supporting Information [file ADVS-7-2000804-s001.pdf]

© 2020 Wiley-VCH GmbH

ADVANCED SCIENCE
Open Access

Supporting Information

for *Adv. Sci.*, DOI: 10.1002/advs.202000804

Axially chiral biphenyl compound-based thermally activated delayed fluorescent materials for high-performance circularly polarized organic light-emitting diodes

Zhen-Long Tu, Zhi-Ping Yan, Xiao Liang, Lei Chen, Zheng-Guang Wu, Yi Wang, You-Xuan Zheng, Jing-Lin Zuo, Yi Pan*

Copyright WILEY-VCH Verlag GmbH & Co. KGaA, 69469 Weinheim, Germany, 2018.

Supporting Information

Axially chiral biphenyl compound-based thermally activated delayed fluorescent materials for high-performance circularly polarized organic light-emitting diodes

Zhen-Long Tu, Zhi-Ping Yan, Xiao Liang, Lei Chen, Zheng-Guang Wu, Yi Wang, You-Xuan Zheng, Jing-Lin Zuo, Yi Pan*

Mr. Z.-L. Tu, Mr. Z.-P. Yan, Mr. X. Liang, Mr. L. Chen, Dr. Z.-G. Wu, Dr. Y. Wang, Prof. Y.-X. Zheng , Prof. J.-L. Zuo, Prof. Y. Pan
State Key Laboratory of Coordination Chemistry, Collaborative Innovation Center of Advanced Microstructures, Jiangsu Key Laboratory of Advanced Organic Materials, School of Chemistry and Chemical Engineering, Nanjing University, Nanjing, 210023, P. R. China
E-mail: yxzheng@nju.edu.cn

1. General information

1.1 Materials, instruments, measurement and DFT calculations

All synthesis experiments were performed under nitrogen atmosphere and final products were purified by sublimation under the pressure of 5×10^{-5} Pa at 230 ^{o}C.

NMR measurements were conducted on a Bruker AM 400 spectrometer. Absorption and emission spectra were measured on a UV-3100 and a Hitachi F-4600 photoluminescence spectrophotometer, respectively. The absolute photoluminescence quantum yield and the decay lifetime of the compound were measured with HORIBA FL-3 fluorescence spectrometer. Temperature-dependent transient PL decay curve was measured with Edinburgh FLS920 fluorescence spectrometer. Thermogravimetric analysis was performed on a Pyris 1 DSC under nitrogen at a heating rate of 10 □ min^{-1}. (*R*)-BPPOACZ and (*S*)-BPPOACZ were separated by column (IG-3 (IG30CD-WE016)) which was employed as stationary phase and hexane/dichloromethane/diethylamine (60/40/0.1) (V/V/V) as eluent with enantiomeric excesses (ee) > 99%. The ECD spectra were measured on a Jasco J-810 circular dichroism spectrometer. CPL and CPEL spectra were performed with a JASCO CPL-300 spectrometer.

Cyclic voltammetry measurement was conducted on an MPI-A multifunctional

electrochemical and chemiluminescent system, with a polished Pt plate working electrode, platinum thread counter electrode and Ag-$AgNO_3$ (0.1 M) in CH_3CN as reference electrode, *tetra*-n-butylammonium perchlorate (0.1 M) as the supporting electrolyte, using Fc^+/Fc as the internal standard. Oxidation potentials was investigated at room temperature with a scan rate of 100 mV/s and HOMO energy was calculated from the oxidation potential with the formula of HOMO = -[E_{ox} - $E_{(Fc/Fc+)}$ + 4.8] eV. The energy gap (E_g) of HOMO and LUMO was calculated from the onset of the absorption spectrum with the formula of E_g, = 1240 / λ_{onset} and LUMO energy was calculated from HOMO - E_g. Density functional theory (DFT) calculations: The ground state geometries of (*R*)-BPPOACZ are optimized by DFT using the B3LYP functional. In order to investigate the energies and the transition characters of the low-lying excited singlet and triplet states, time-dependent DFT (TD-DFT) with B3LYP functional was used to calculate the vertical absorption energies for S_1, T_1 and ΔE_{ST}. All the above calculations were carried out by using Gaussian 09 software with the 6-31G basis set (Ref. Gaussian 09, Revision D.01, M. J. Frisch, *et al.*, Gaussian, Inc., Wallingford CT, 2013). The functions of reduced density gradient (RDG) and Sign $(\lambda_2)\rho$ were calculated using Multiwfn software (Ref. T. Lu *et al.*, *J. Comput. Chem.*, **2012**, *33*, 580-592).

1.2 Fabrication and measurement of CP-OLEDs

Indium-tin-oxide (ITO) coated glass with a sheet resistance of 10 Ω sq^{-1} was used as the anode substrate. Prior to film deposition. All the organic layers were deposited with the rate of 0.1 nm s^{-1} under high vacuum ($\leq 2\times10^{-5}$ Pa). The doped layers were prepared by co-evaporating dopant and host material from two individual sources, and the doping concentrations were modulated by controlling the evaporation rate of dopant. LiF and Al were deposited in another vacuum chamber ($\leq 8.0\times10^{-5}$ Pa) with the rates of 0.01 and 1 nm s^{-1}, respectively, without being exposed to the atmosphere. Device performances were measured by using a programmable Keithley source measurement unit with a silicon photodiode. The EL spectra were measured with a calibrated Hitachi F-7000 fluorescence spectrophotometer. Based on the uncorrected EL fluorescence spectra, the Commission Internationale de l'Eclairage (CIE) coordinates were calculated using the test program of Spectrascan PR650 spectrophotometer. The EQEs of EL devices were calculated based on the photo energy measured by the photodiode, the EL spectrum and the current pass through the device. The circularly polarized electroluminescence (CPEL) spectra were measured on a Jasco CPL-300 spectrophotometer.

1.3 Measurement of the chiroptical properties in thin film and the g_{EL} of the device

Firstly, thin films and devices were fabricated through vacuum evaporation in same conditions, for example under the pressure of 2×10^{-5} Pa and at the temperature of 195 ^{o}C for BPPOACZ. Secondly, all CPL and CPEL characteristics were measured by using JASCO CPL-300. Thin films are small enough to be sandwiched in CHUCKING APPLIANCE and no adjustment was necessary during the measurements to keep the light path parallel to avoid refraction. A distance of 5.5 cm between sample and EXCITATION can achieve high signal to noise. Devices were too high from the light path when sandwiched in CHUCKING APPLIANCE, so appropriate adjustments were needed. A 5.5 cm wide hard card was abutted against the side of EXCITATION. Then an UNFIXED SLIDE was lowered the height and abutting against the other side of the hard card. And the devices could be sandwiched in CHUCKING APPLIANCE as high as the light path. After a baffle covering the xenon lamp from EXCITATION, devices could be turned on with an external power. All contacts during the adjustment were very tight to keep light path parallel. Thirdly, the CPPL spectra were measured on a Jasco CPL-300 spectrophotometer with “Standard” sensitivity at 200 nm/min scan speed and respond time of 2.0 s employing “slit” mode. Following are photos and sketches of the measurements.

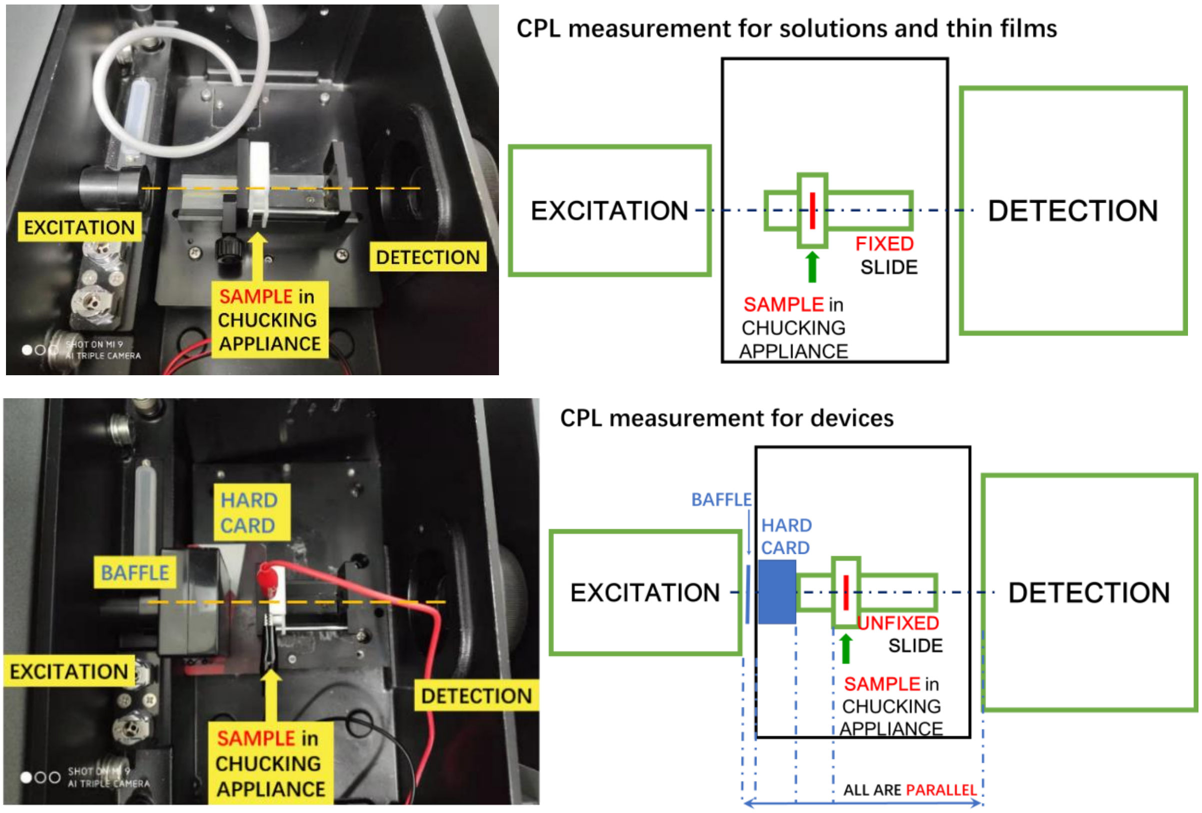

Figure S1. Photos and sketches of measurement of the chiroptical properties in thin film and the g_{EL} of the device.

2. Synthetic procedures and characterized data

2.1 Synthesis routes and procedures for (*rac*)-BPPOACZ

The synthesis route of (*rac*)-BPPOACZ is shown in Scheme S1.

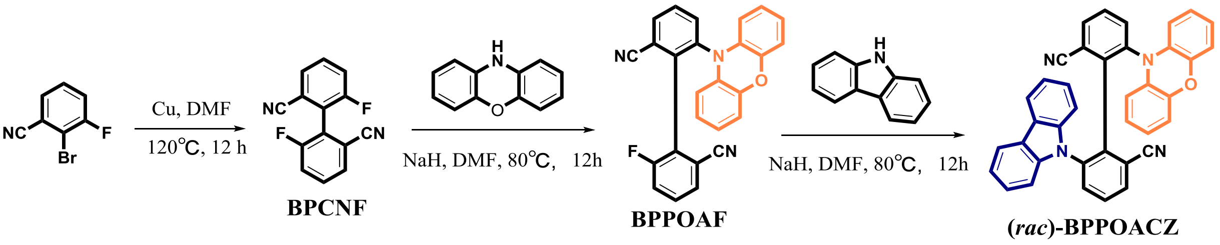

Scheme S1. Synthesis route of (*rac*)-BPPOACZ.

2.1.1 Preparation procedure for BPCNF

The solution of 2-bromo-3-fluorobenzonitrile (4.00 g, 20.0mmol), copper powder (1.27g, 20.0 mmol) in dry DMF (20 mL) was stirred for 12 h at 120℃. After cooling down to room temperature, the suspension mixture was poured into water (100 mL), and the aqueous layer was extracted with dichloromethane (3×20 mL), which was washed with water and concentrated under vacuum. The product was the purified by flash chromatography (EA/PE, 1:6) to get the white solid 1.87 g (78%). ^{1}H NMR (400 MHz, $CDCl_3$) δ 7.67 (dd, *J* = 7.7, 1.4 Hz, 2H), 7.63 (td, *J* = 7.9, 4.7 Hz, 2H), 7.52 (td, *J* = 8.7, 1.5 Hz, 2H). ^{19}F NMR (376 MHz, $CDCl_3$) δ -108.95 (s). ^{13}C NMR (101 MHz, $CDCl_3$) δ 159.83 (d, *J* = 253.4 Hz), 132.33 (d, *J* = 9 Hz), 132.32 (d, *J* = 2.9 Hz), 129.41 (t, *J* = 1.6 Hz), 123.27 (d, *J* = 19.7 Hz), 121.26 (d, *J* = 23.4 Hz), 115.79 (d, *J* = 50.2 Hz).

2.1.2 Preparation procedure for BPPOAF

The suspension mixture of phenoxazine (1.0 g, 5.5 mmol) and sodium hydride (242 mg, 6.05 mmol) in dry DMF (30 mL) was stirred for 0.5 h at room temperature. After adding BPCNF (1.20 g, 5 mmol), the mixture was heated at 80 □ for 12 h, and then was poured into water (100 mL). The aqueous layer was extracted with ethyl acetate (3×20 mL), and the combined organic phase was washed with water (50 mL) and concentrated under vacuum. The crude product was the purified by flash chromatography (EA/PE, 1:6) to get the yellow solid 1.25 g (62%). ^{1}H NMR (400 MHz, $CDCl_3$) δ 7.98 (dd, *J* = 5.4, 3.7 Hz, 1H), 7.87 - 7.86 (m, 2H), 7.46 - 7.42 (m, 2H), 7.29 (ddd, *J* = 9.5, 6.9, 2.7 Hz, 1H), 6.71 - 6.67 (m, 5H), 6.64 - 6.61 (m, 2H), 6.13 (ddd, *J* = 5.1, 3.0, 1.2 Hz, 2H). ^{19}F NMR (376 MHz, $CDCl_3$) δ -107.28 (s).

^{13}C NMR (101 MHz, $CDCl_3$) δ 159.83 (d, J = 251.8 Hz), 144.13 (s), 140.43 (s), 137.84 (s), 137.41 (s), 133.40 (s), 132.88 (s), 132.77 (s), 131.90 (d, J = 8.9 Hz), 129.26 (d, J = 3.7 Hz), 125.85 (d, J = 19.7 Hz), 123.45 (s), 122.67 (s), 120.85 (d, J = 22.2 Hz), 117.25 (s), 116.30 (d, J = 4.0 Hz), 116.20 (s), 115.83 (s), 115.49 (d, J = 4.0 Hz), 114.28 (s). MALDI-TOF: Calculated for $C_{26}H_{14}FN_3O$: 403.1121, Found: 403.2904.

2.1.3 Preparation procedure for (*rac*)-BPPOACZ

The suspension mixture of carbazole (551.8 mg, 3.3 mmol), and sodium hydride (145 mg, 3.63 mmol) in dry DMF (15 mL) was stirred for 0.5 h at room temperature. After adding BPPOAF (1.25 g, 3 mmol), the mixture was stirred for 12 h at 80 □ and poured into water (60 mL), which was extracted with ethyl acetate (3×10 mL). The organic phase was washed with water (30 mL) and concentrated under vacuum. The crude product was purified by flash chromatography (EA/PE, 1:6) to get the pale yellow solid 1.14 g (69%). The pale yellow solid was purified by sublimation to get pale yellow crystal (50%) and then the crystal was improved purity by sublimation with 90% yield. ^{1}H NMR (400 MHz, $CDCl_3$) δ 8.00 (dd, J = 7.7, 1.3 Hz, 1H), 7.89 - 7.85 (m, 2H), 7.65 (dd, J = 8.2, 1.4 Hz, 2H), 7.61 (t, 2H), 7.50 (dd, J = 8.2, 1.2 Hz, 1H), 7.30 - 7.25 (m, 1H), 7.22 - 7.10 (m, 4H), 6.99 (t, J = 7.9 Hz, 1H), 6.73 - 6.67 (m, 3H), 6.24 (dtd, J = 9.6, 7.9, 1.5 Hz, 2H), 6.02 - 5.97 (m, 1H), 5.75 (ddd, J = 8.1, 7.3, 1.7 Hz, 1H), 4.61 (dd, J = 8.1, 1.1 Hz, 1H). ^{13}C NMR (101 MHz, $CDCl_3$) δ 143.92 (s), 143.13 (s), 141.96 (s), 140.50 (s), 140.44 (s), 140.36 (s), 138.94 (s), 136.48 (s), 136.27 (s), 134.59 (s), 133.93 (s), 133.41 (s), 133.36 (s), 132.98 (s), 132.35 (s), 131.26 (s), 126.58 (s), 125.70 (s), 124.29 (s), 124.17 (s), 123.46 (s), 122.65 (s),122.50 (s), 122.42 (s), 120.65 (s), 120.40 (s), 120.18 (s), 119.82 (s), 118.03 (s), 117.72 (s), 117.57 (s) 116.97 (s), 115.71 (s), 115.34 (s), 113.83 (s), 112.31 (s), 110.05 (s), 109.49 (s). MALDI-TOF: Calculated for $C_{38}H_{22}N_4O$: 550.1793, Found: 550.5169. Anal. calcd for C38H22N4O: C, 82.89; H, 4.03; N, 10.18; found: C,82.61; H, 4.00; N, 10.18.

3. Prior literature examples of CPL active TADF materials.

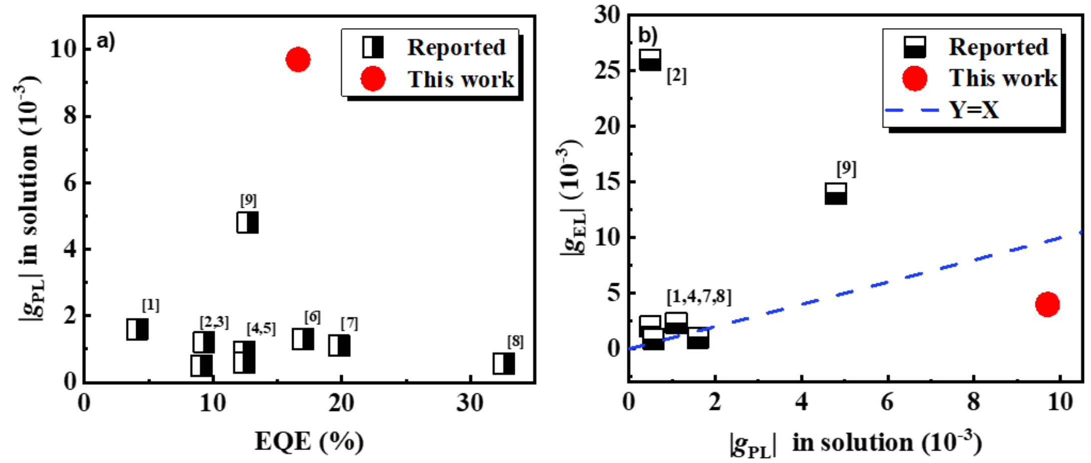

Figure S2. a) $|g_{PL}|$ of CP-TADF materials in solutions versus highest external quantum efficiency in corresponding devices; b) $|g_{EL}|$ of CP-OLEDs versus $|g_{PL}|$ in solutions of corresponding CP-TADF materials.

4. Copies of ^{1}H NMR, ^{19}F NMR, ^{13}C NMR and MALDI-TOF spectra of new compounds.

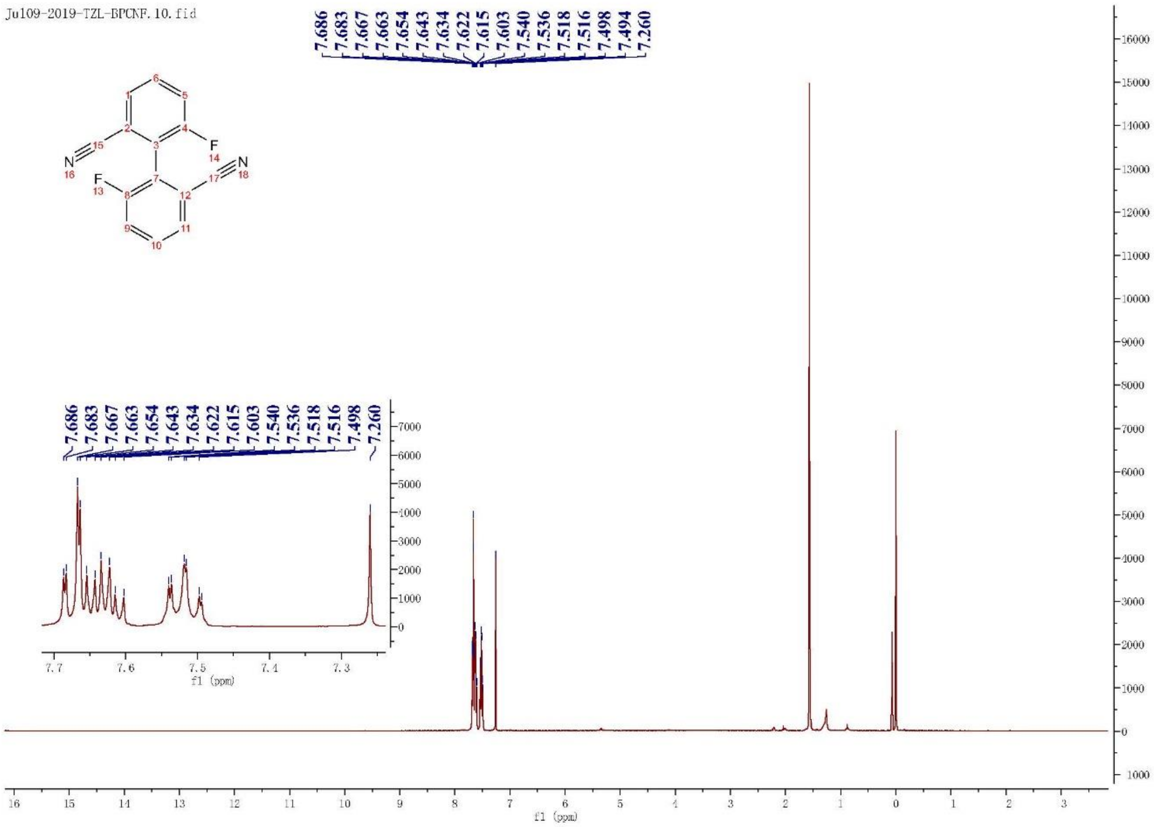

Figure S3-1. ^{1}H NMR (400 MHz, $CDCl_3$) spectrum of BPCNF.

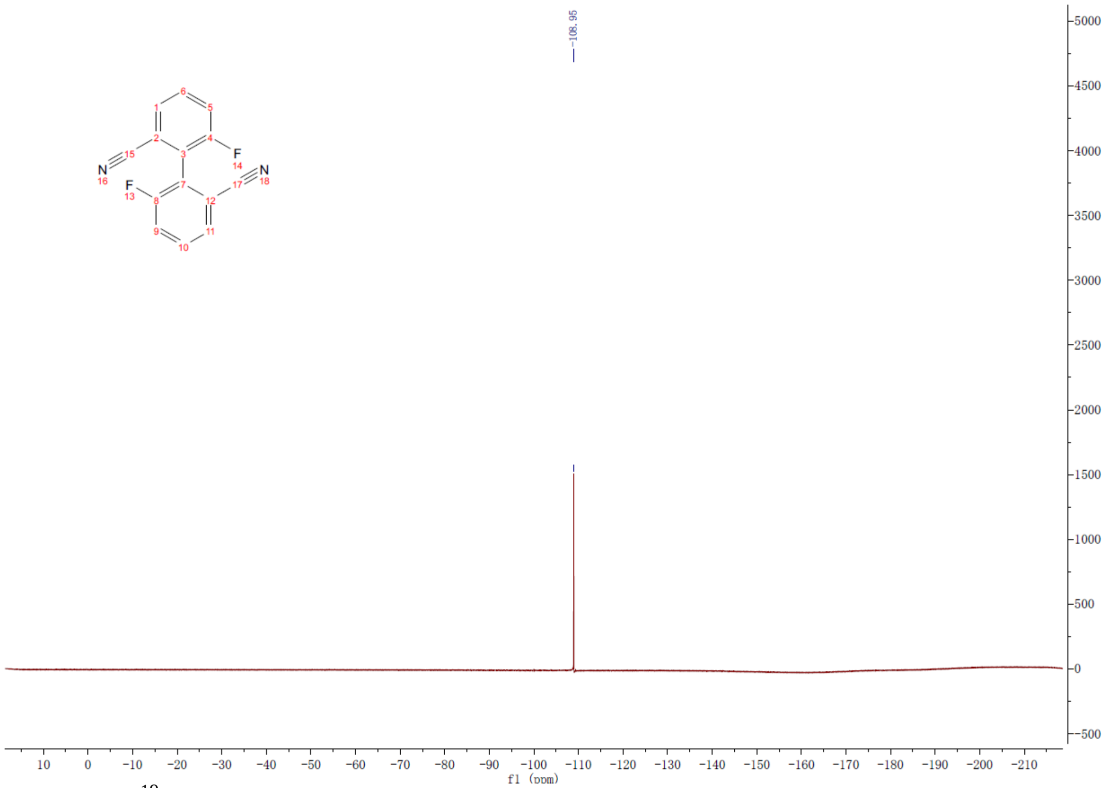

Figure S3-2. ^{19}F NMR (376 MHz, $CDCl_3$) spectrum of BPCNF.

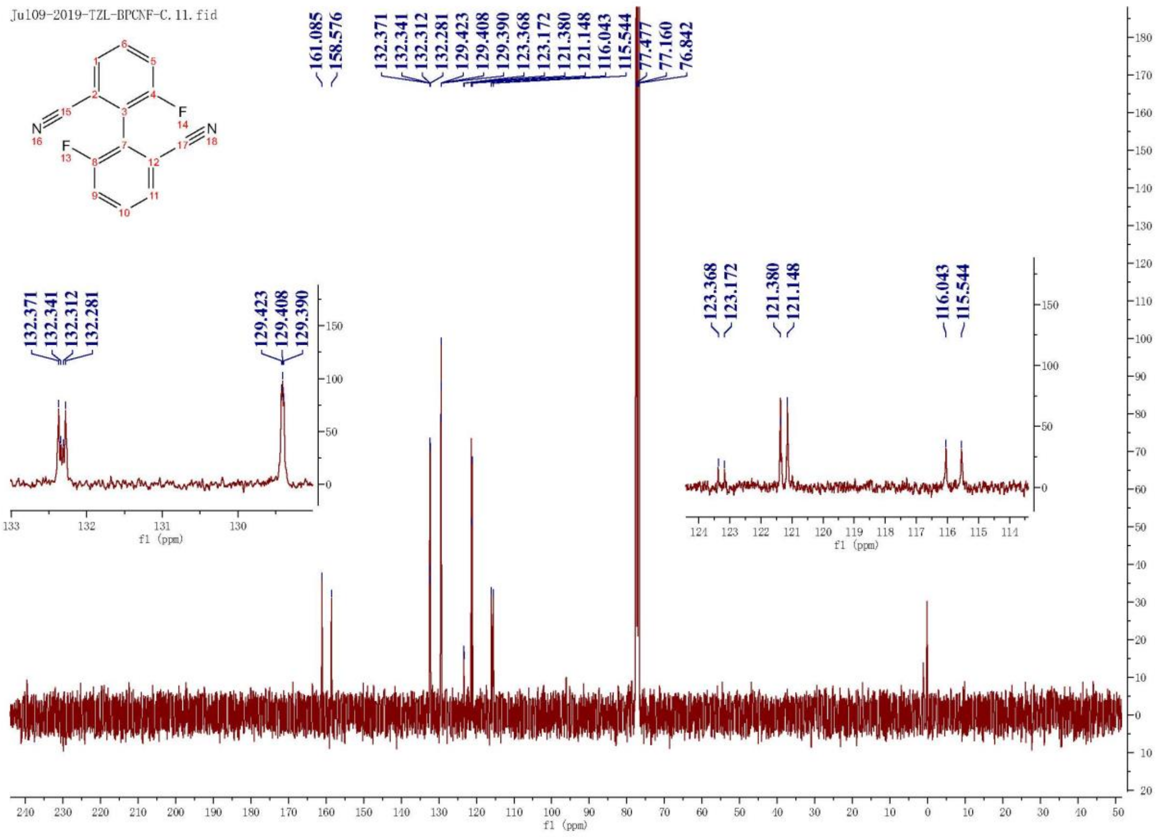

Figure S3-3. ^{13}C NMR (101 MHz, $CDCl_3$) spectrum of BPCNF.

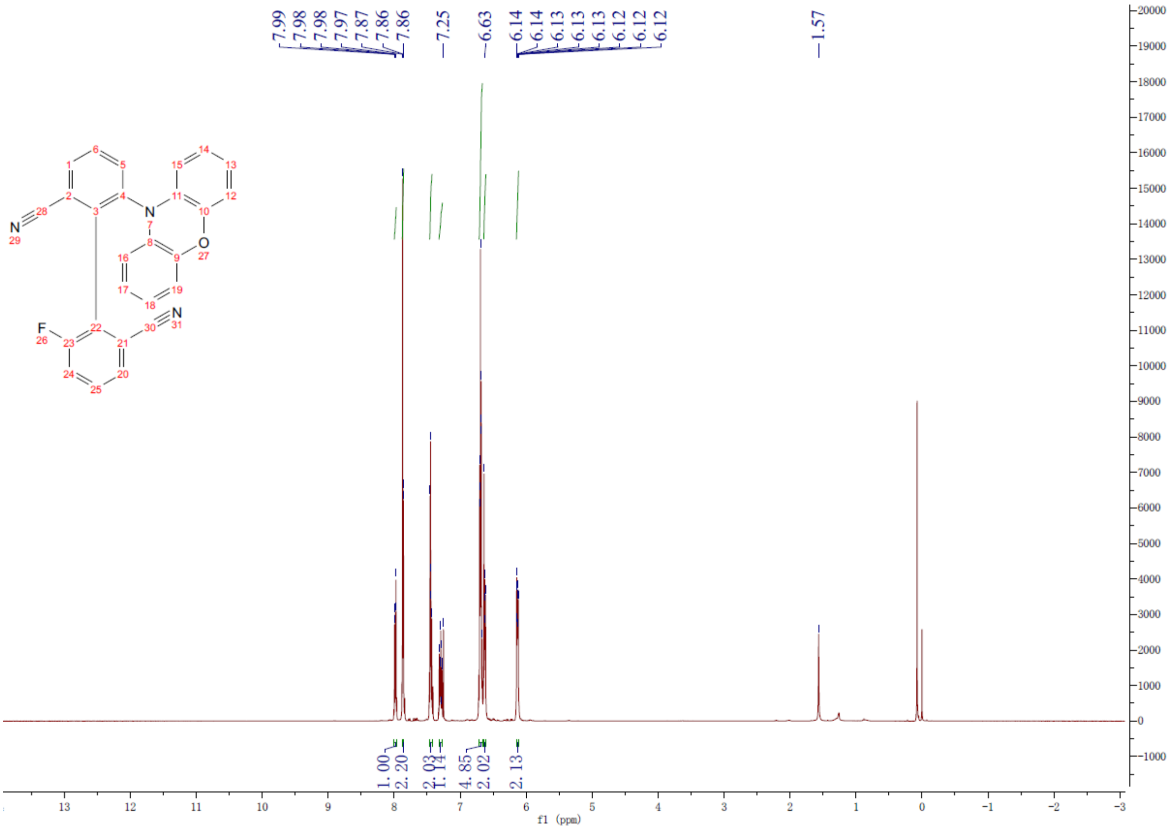

Figure S3-4. ^{1}H NMR (400 MHz, $CDCl_3$) spectrum of BPPOAF.

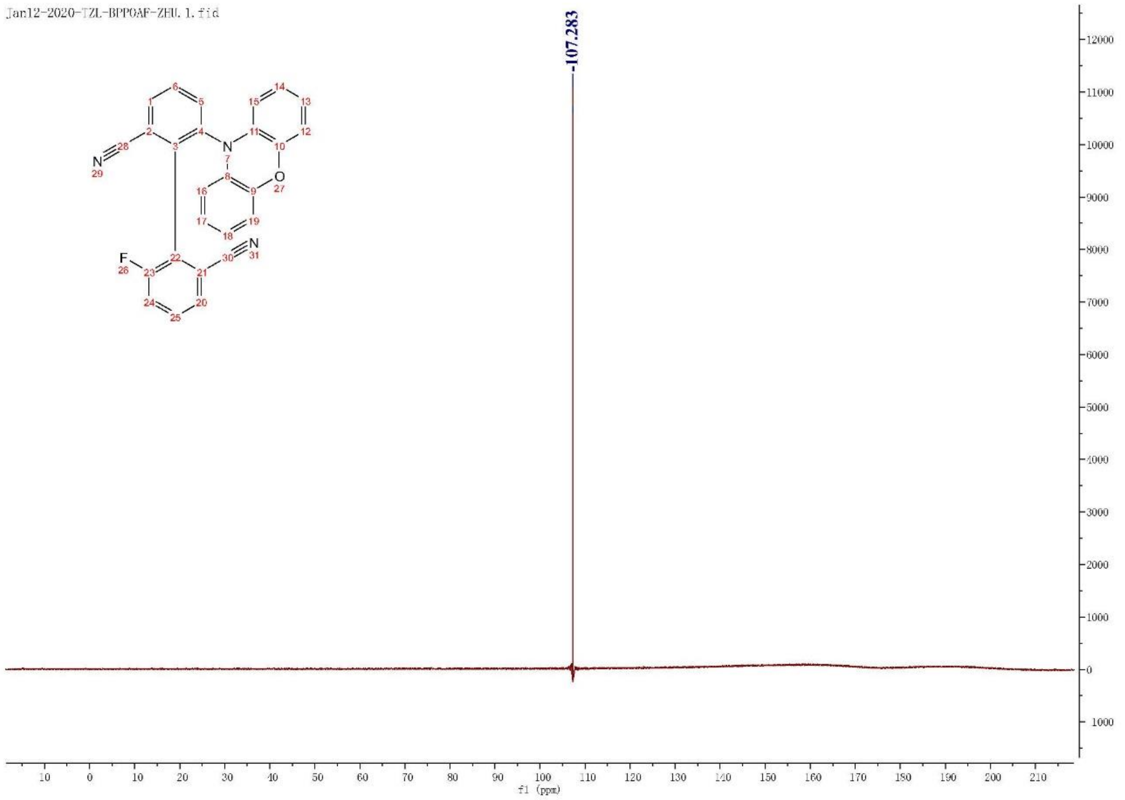

Figure S3-5. ^{19}F NMR (376 MHz, $CDCl_3$) spectrum of BPPOAF.

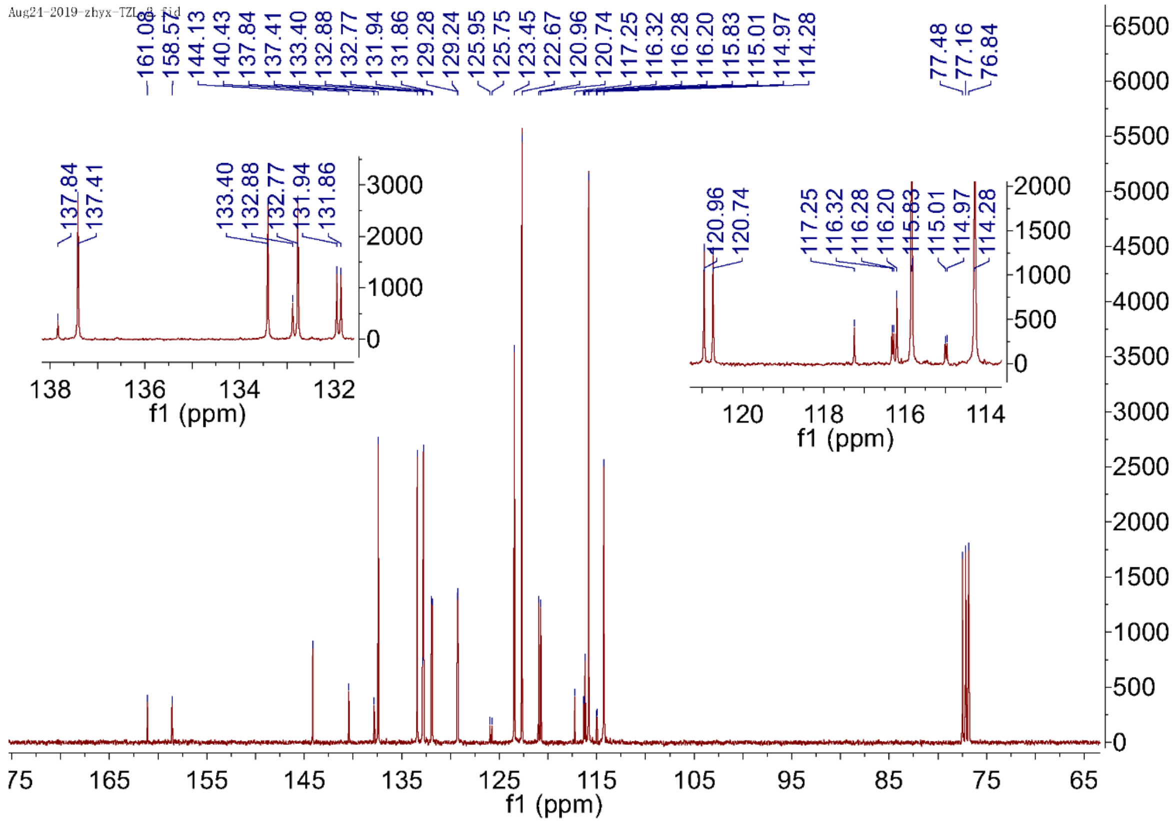

Figure S3-6. ^{13}C NMR (101 MHz, $CDCl_3$) spectrum of BPPOAF.

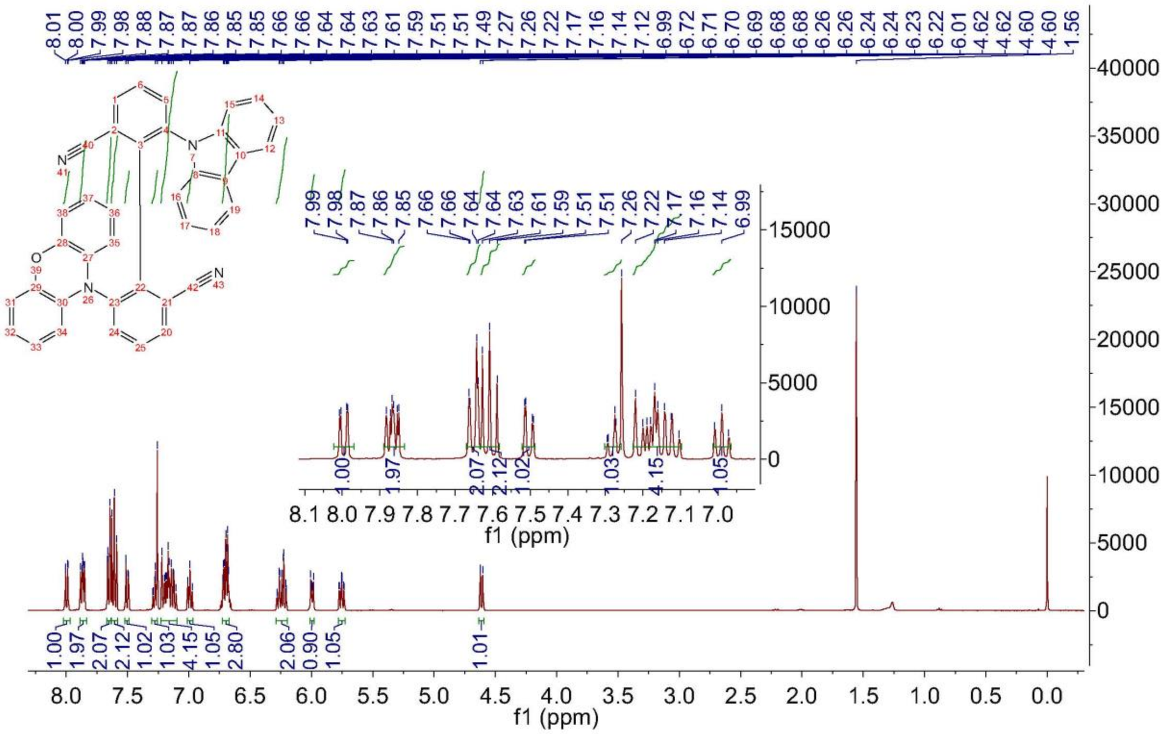

Figure S3-7. ^{1}H NMR (400 MHz, $CDCl_3$) spectrum of BPPOACZ.

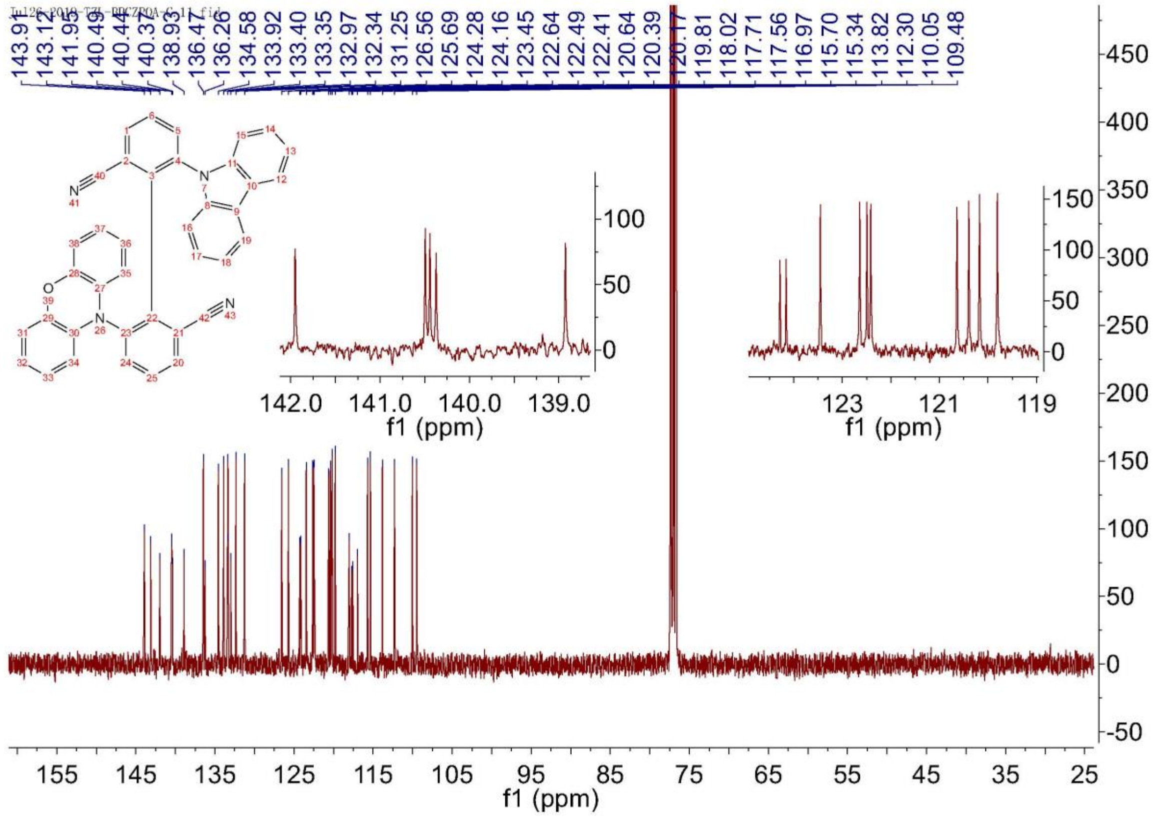

Figure S3-8. ^{13}C NMR (101 MHz, $CDCl_3$) spectrum of BPPOACZ.

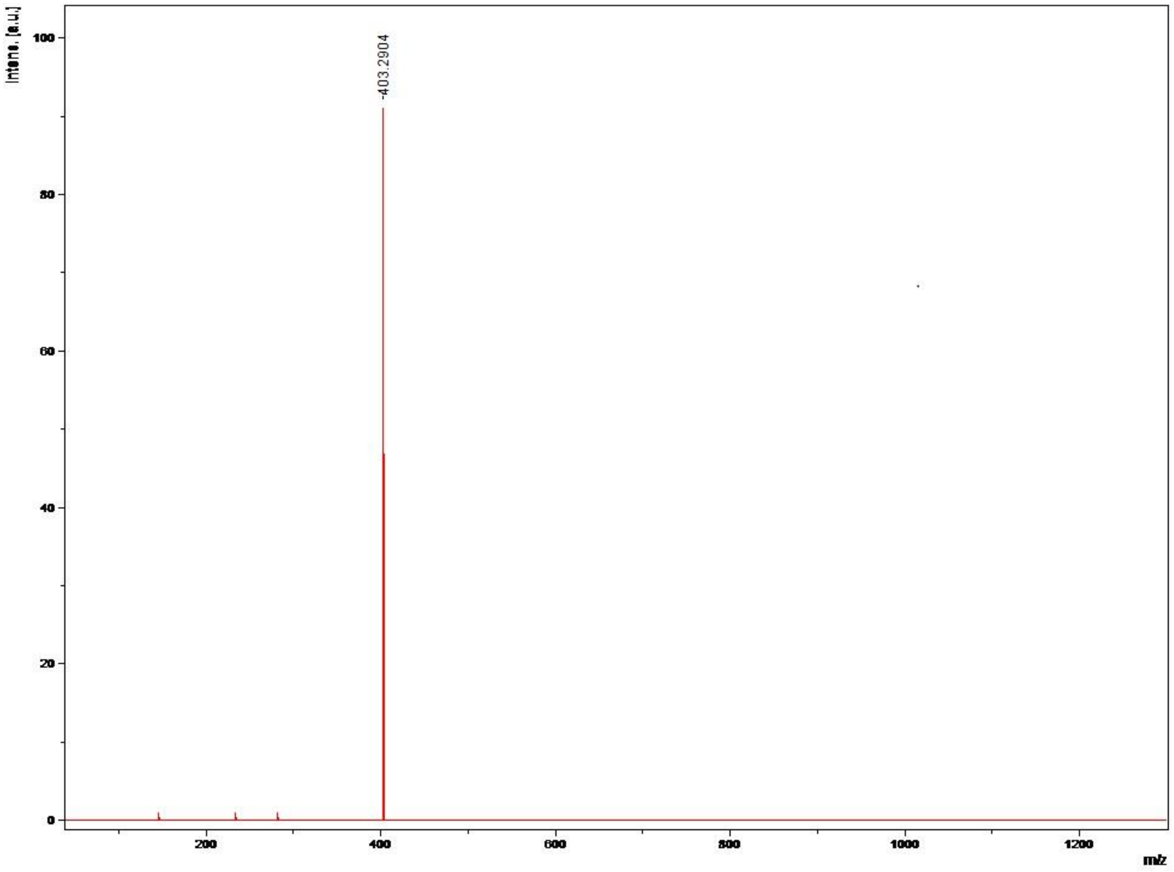

Figure S3-9. MALDI-TOF spectrum of BPPOAF.

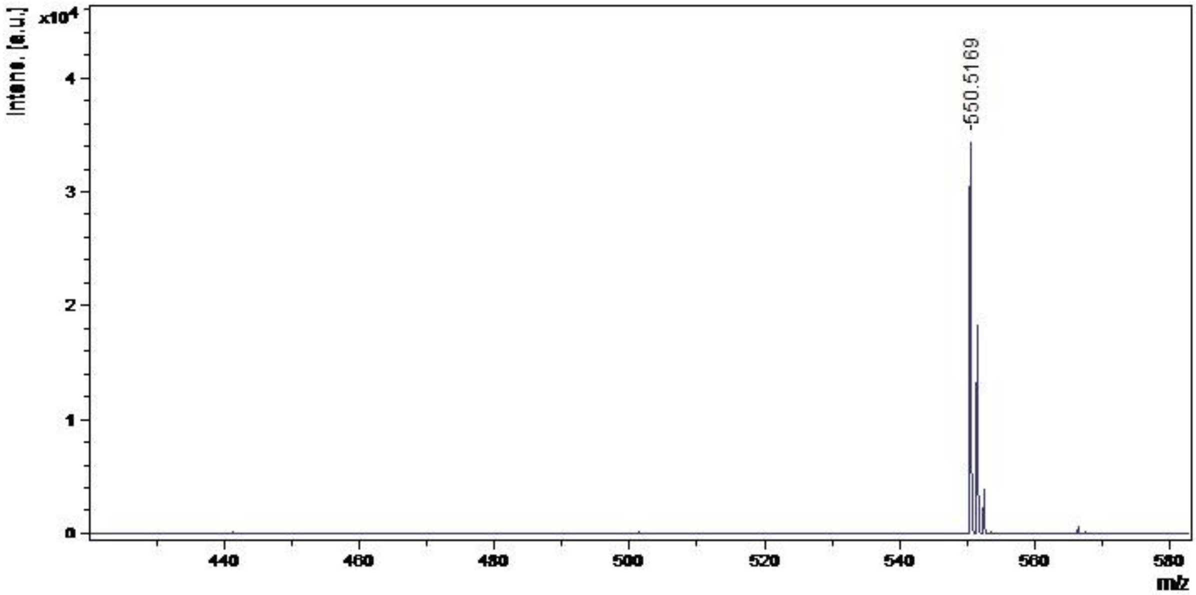

Figure S3-10. MALDI-TOF spectrum of BPPOACZ.

5. Chiral HPLC Analysis

HPLC Analysis Conditions: a) Column: IG-3(IG30CD-WE016), 2.0 ul, 0.46 cm I.D. ×15 cm L; b) Mobile phase: Hexane/DCM/DEA=60/40/DEA(V/V/V); c) Flow rate: 1.0 mL/min; d) Abs. detector: 254 nm.

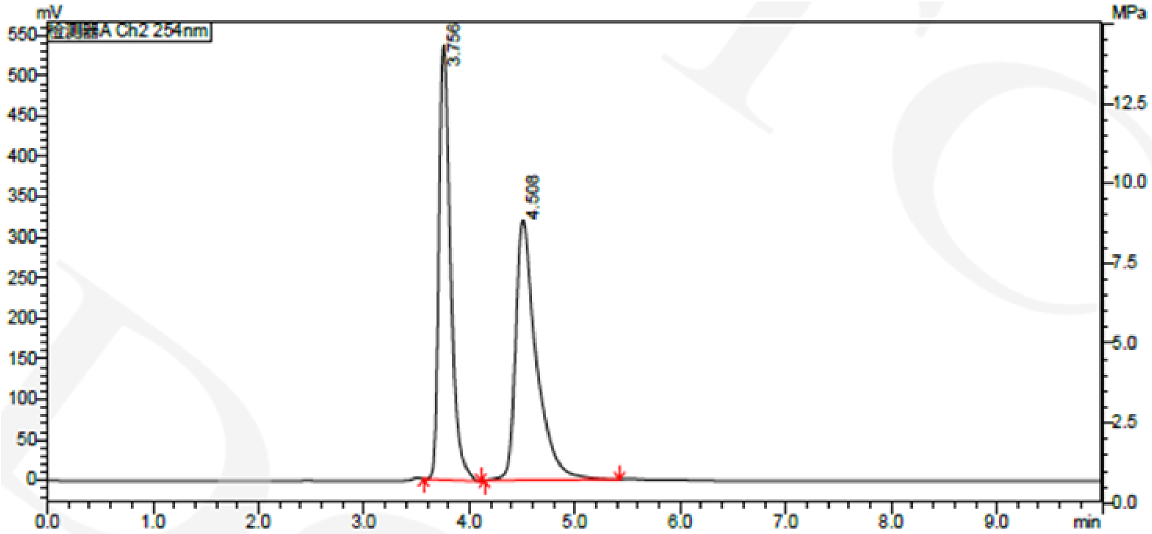

<Peak Table>

Peak#	Ret. Time	Area	Area%	T.Plate#	Tailing F.	Resolution
1	3.756	4103450	48.364	5399	1.464	--
2	4.508	4381078	51.636	2974	1.732	2.808

Figure S4-1. HPLC profile of (*rac*)-BPPOACZ.

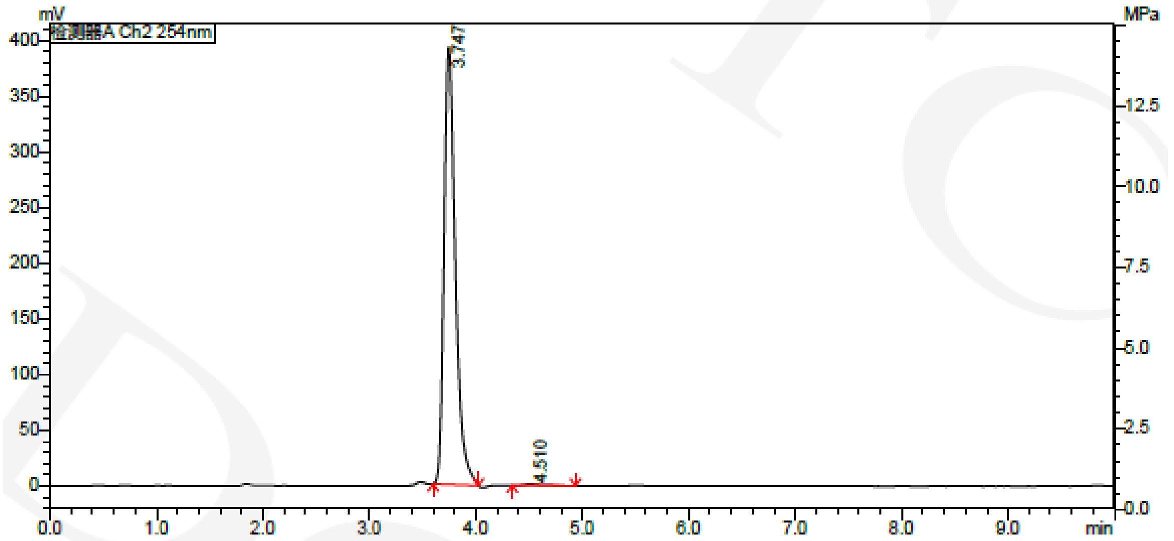

<Peak Table>

Peak#	Ret. Time	Area	Area%	T.Plate#	Tailing F.	Resolution
1	3.747	2896099	99.422	5649	1.427	--
2	4.510	16840	0.578	1178	1.818	2.105

Figure S4-2. HPLC profile of (*S*)-BPPOACZ.

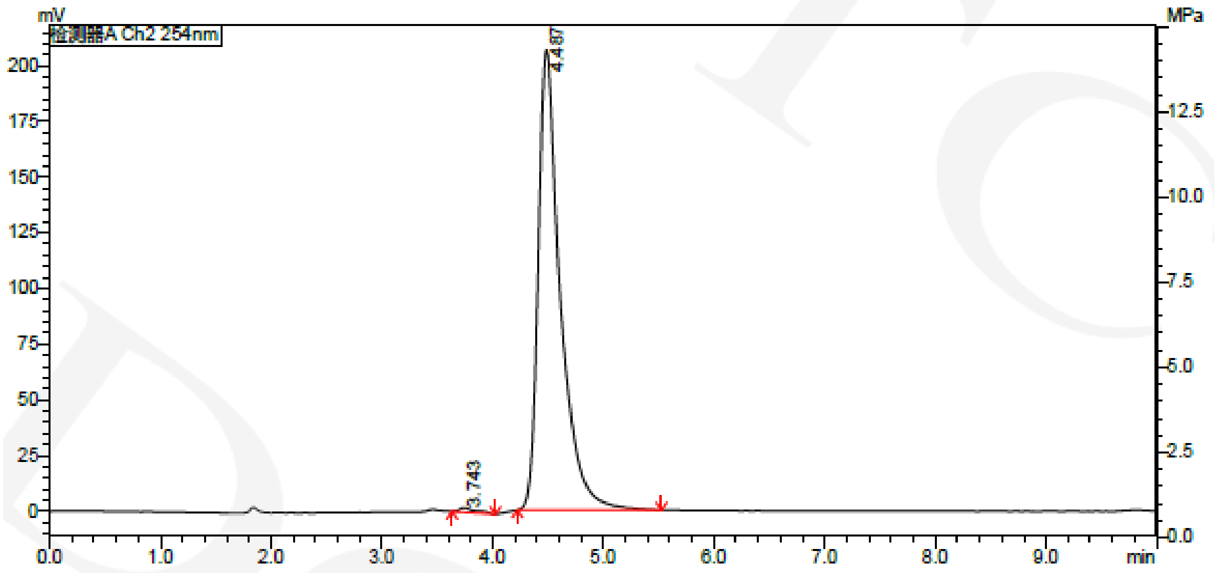

<Peak Table>

Peak#	Ret. Time	Area	Area%	T.Plate#	Tailing F.	Resolution
1	3.743	20527	0.714	4489	1.837	--
2	4.487	2854607	99.286	2851	1.644	2.659

Figure S4-3. HPLC profile of (*R*)-BPPOACZ.

Table S1. Summary of steps of gradient sublimation.

Step	Time (s)	Preserved Temperature1 (°C)	Preserved Temperature2 (°C)	Sublimation Temperature (°C)
1	60	30	30	30
2	1800	100	120	130
3	2400	100	135	170
4	2400	110	150	190
5	1800	120	160	200
6	5400	140	185	230
7	65535(max)	140	185	230
8	65535(max)	140	185	230

During the sublimation of BPPOACZ, the pressure could reach 5 × 10^{-5} Pa and the final temperature could reach 230 °C. The "gradient sublimation" means that the speed of rising-temperature decreases as the temperature increases. The procedure was summarized in the table and rising-temperature kept constant speed in every step.

6. Crystal data and structure.

Table S2. Crystal data and structure refinement for (*R*)-BPPOACZ.

Identification code	(*R*)-BPPOACZ
Empirical formula	$C_{38}H_{22}N_4O$
Formula weight	550.60
Temperature/K	210.02
Crystal system	orthorhombic
Space group	$P2_12_12_1$
a/Å	8.1095(5)
b/Å	9.4050(5)
c/Å	37.327(2)
α/°	90.00
β/°	90.00
γ/°	90.00
Volume/Å^3	2846.9(3)
Z	4
$\rho_{calc}g/cm^3$	1.285

μ/mm^{-1}	0.400
F(000)	1144.0
Crystal size/mm^3	0.11 × 0.09 × 0.08
Radiation	GaKa (λ = 1.34139)
2Θ range for data collection/°	4.12 to 106
Index ranges	-9 ≤ h ≤ 9, -11 ≤ k ≤ 10, -42 ≤ l ≤ 44
Reflections collected	29338
Independent reflections	5031 [R_{int} = 0.0805, R_{sigma} = 0.0564]
Data/restraints/parameters	5031/780/397
Goodness-of-fit on F^2	1.176
Final R indexes [I>=2σ (I)]	R_1 = 0.1025, wR_2 = 0.1936
Final R indexes [all data]	R_1 = 0.1506, wR_2 = 0.2167
Largest diff. peak/hole / e Å^{-3}	0.33/-0.51
CCDC	1962429

$R_1{}^a = \Sigma||F_o| - |F_c||/\Sigma F_o|$. $wR_2{}^b = [\Sigma w(F_o{}^2 - F_c{}^2)^2/\Sigma w(F_o{}^2)]^{1/2}$

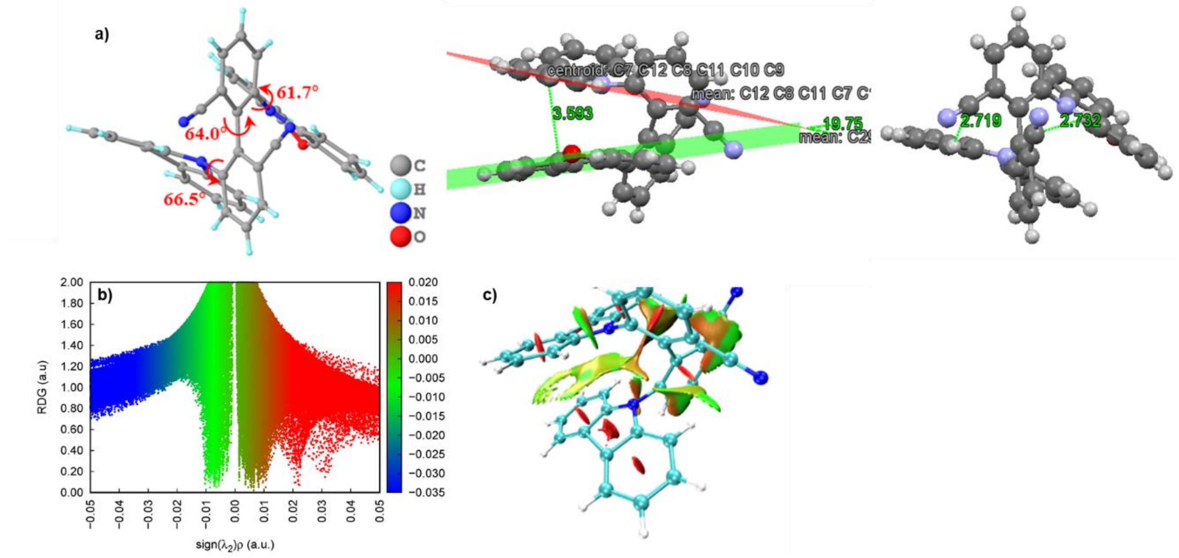

Figure S5. a) Single crystal of (*R*)-BPPOACZ; b) The functions of reduced density gradient (RDG) and Sign (λ_2)ρ for (*R*)-BPPOACZ; c) RDG isosurface map with an isovalue of 0.55 for (*R*)-BPPOACZ.

7. TGA Measurement

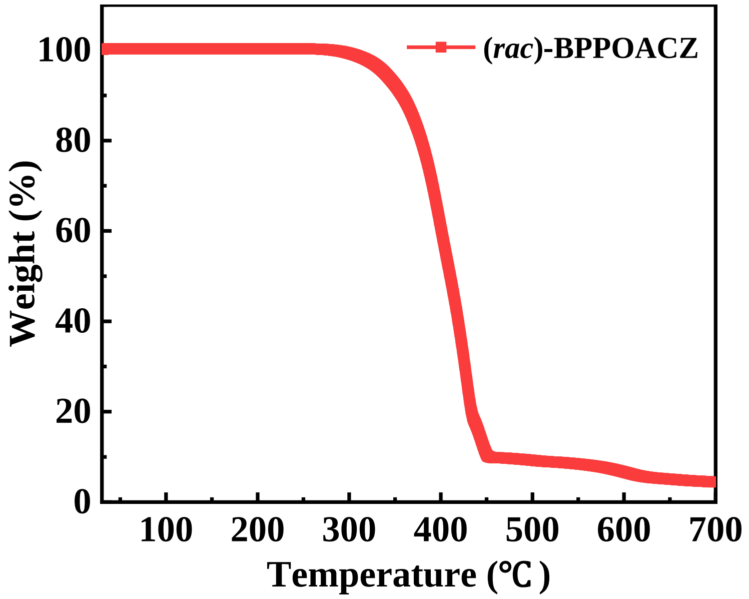

Figure S6. TGA curves of (*rac*)-BPPOACZ.

8. DFT calculations and electrochemical measurement.

Table S3. HOMOs and LUMOs distribution of (*R*)-BPPOACZ and corresponding energies of molecular orbitals.

MO		Composition (%)				Energy[a] (eV)	Energy[b] (eV)
		CN	Biph	Cz	POA		
LUMO+2	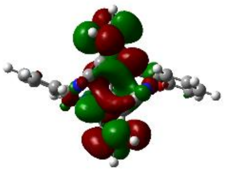	2.72	85.35	5.20	6.73		
LUMO+1	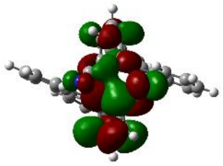	16.51	76.25	3.43	3.82	-1.744	

LUMO	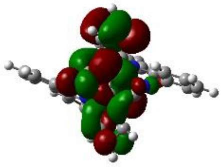	14.75	80.22	2.72	2.31	-1.844	-2.61
HOMO	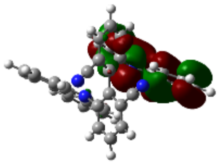	0.11	6.95	1.41	91.53	-5.070	-5.35
HOMO-1	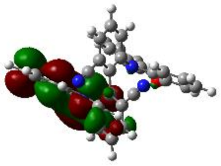	0.26	10.22	88.05	1.47	-5.611	
HOMO-2	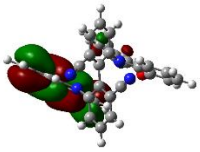	0.09	0.66	95.52	3.73		

[a] Calculated by TD-DFT at B3LYP /6-31g(d) level; [b] Calculated from cyclic voltammetry diagram.

Table S4. Energy levels of S_1 and T_1.

Compound	$E_{VA}(S_1)$[a] [eV]	$E_{VA}(T_1)$[a] [eV]	ΔE_{ST}(vertical)[a] [eV]	$E_{AE}(S_1)$[b] [eV]	$E_{AE}(T_1)$[b] [eV]	ΔE_{ST}(adiabatic)[b] [eV]
(*rac*)-BPPOACZ	2.603	2.551	0.05	2.792	2.756	0.04

[a] The vertical absorption energies of S1 and T1 and the energy gap were calculated by TD-DFT at B3LYP /6-31g(d) level; [b] adiabatic emission energies and energy gap were estimated from the onset of the fluorescence and phosphorescence spectra at 77 K measured in toluene.

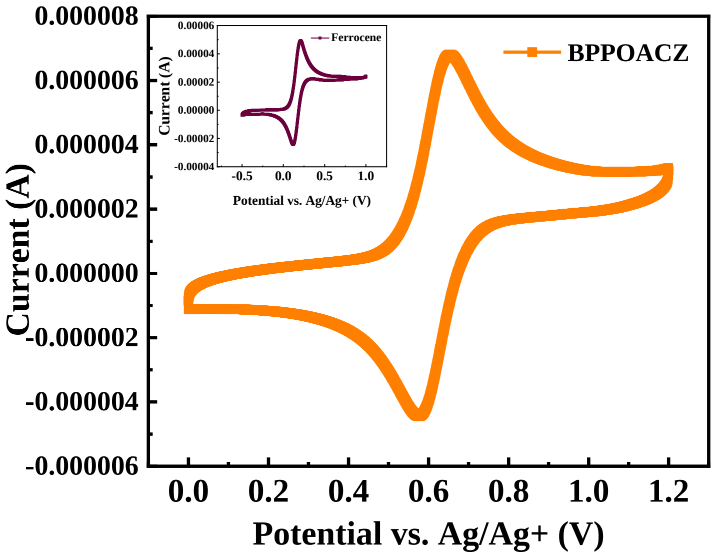

Figure S7. Cyclic voltammetry (CV) diagram of (*rac*)-BPPOACZ in acetonitrile with ferrocene as the internal standard.

9. Photophysical Properties

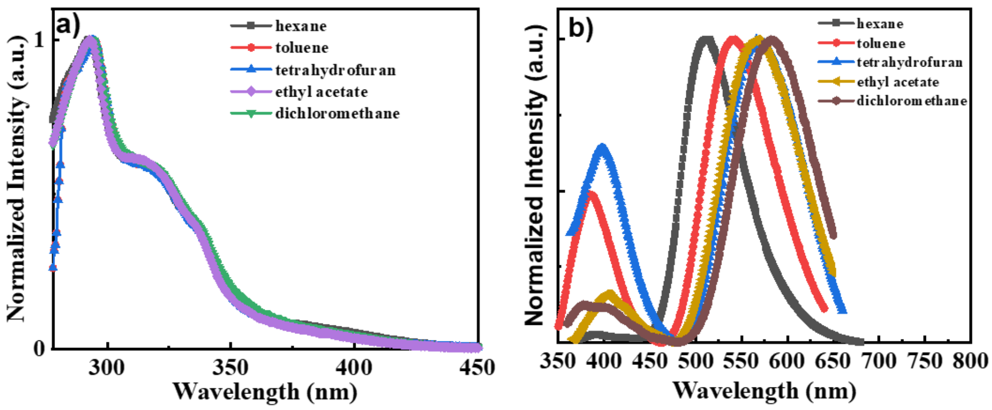

Figure S8. a) Absorption spectra and b) fluorescence spectra of (*rac*)-BPPOACZ in different solvents at room temperature (5.0 × 10^{-5} M).

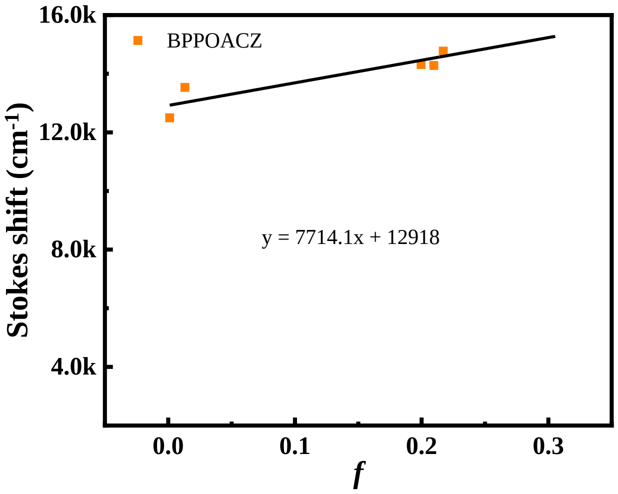

Figure S9. Lippert-Mataga plots for (*rac*)-BPPOACZ.

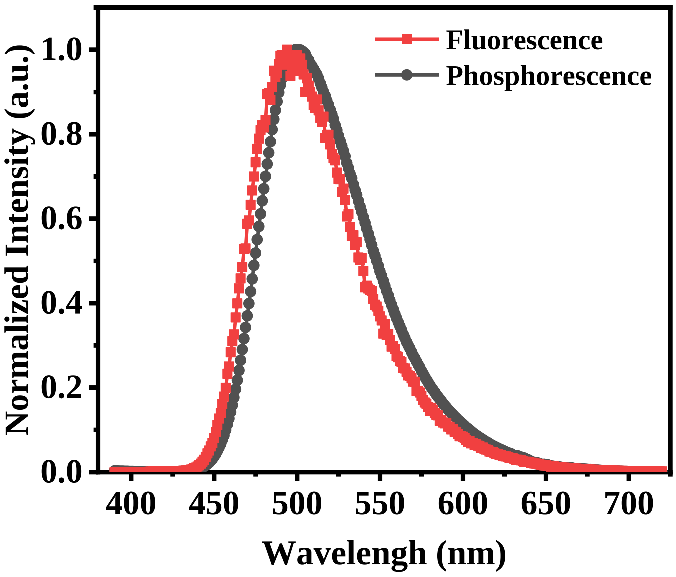

Figure S10. Fluorescence and phosphorescence spectra at 77 K in toluene (5.0 × 10^{-5} M).

Table S5. The UV-vis, PL data and Stokes shift for (*rac*)-BPPOACZ in different solvents.

Solvent [5.0×10^{-5} M]	*E*[a]	n[a]	*f*(ε, n) [a]	$\lambda_{Absorption,max}$[b] [nm]	$\lambda_{emission,max}$[b] [nm]	v_a - v_p[b] [cm^{-1}]
hexane	1.90	1.3749	0.0012	292.5	514.0	14733
toluene	2.38	1.4969	0.0132	294.0	543.0	15597
THF	7.58	1.4072	0.2096	294.0	566.0	16346
ethyl acetate	6.02	1.3724	0.1996	292.5	567.0	16551
CH_2Cl_2	8.93	1.4241	0.2171	294.0	582.0	16831

[a] The ε and n values are obtained from http://www.stenutz.eu/chem/solv23.php. [b] Absorption maximum, fluorescence maximum and Stokes shift (v_a - v_p) of BPPOACZ in different solvents.

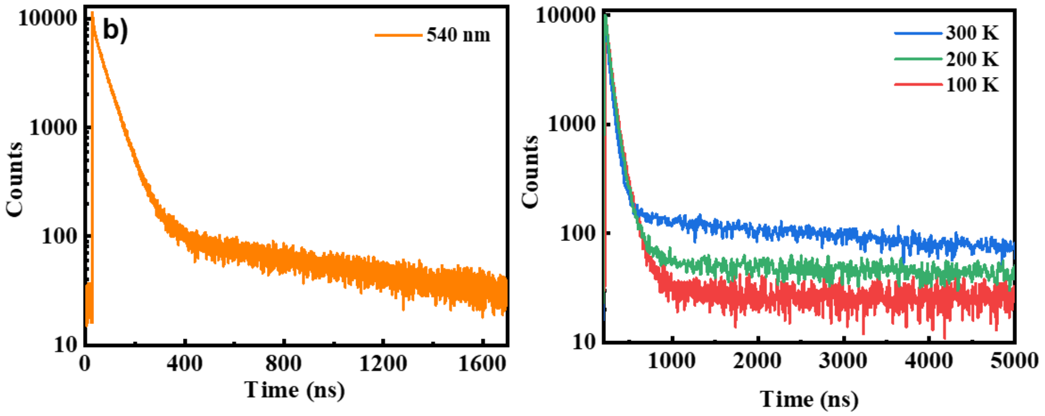

Figure S11. a) PL decay curves of (*rac*)-BPPOACZ in toluene under nitrogen atmosphere at 288 K; b) PL decay curves of powder of (*rac*)-BPPOACZ at 300 K (blue line), 200 K (green line), 100 K (red line).

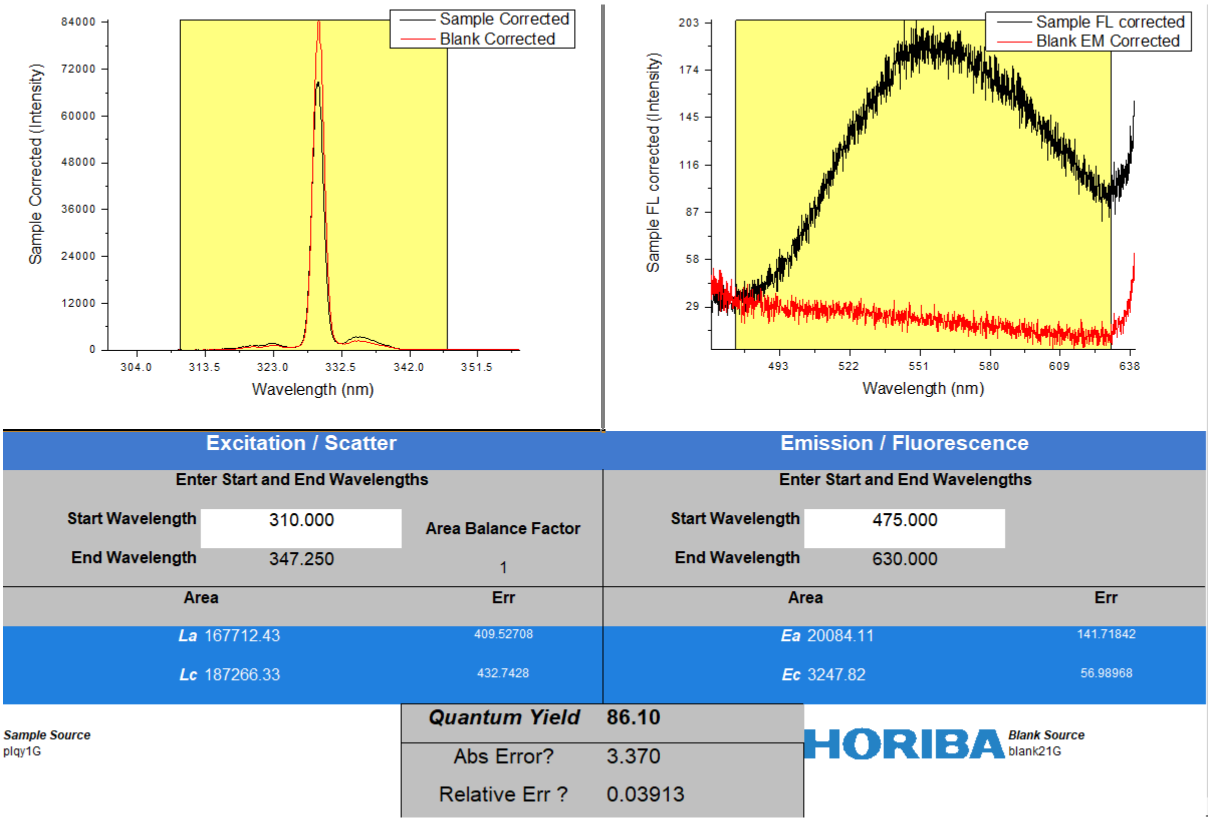

Figure S12. The absolute photoluminescence quantum yields (PLQYs) in toluene solution (5×10^{-5} M) under nitrogen atmosphere determined by an integrating sphere.

Table S6. Photoluminescence properties of (*rac*)-BPPOACZ in toluene under nitrogen atmosphere at room temperature.

Φ[a]	Φ_p[a]	Φ_d[a]	τ_p[b] [ns]	τ_d[b] [μs]	k_p[c] [10^7 s^{-1}]	k_{IC}[c] [10^6 s^{-1}]	k_{ISC}[c] [10^6 s^{-1}]	Φ_{IC}	Φ_{ISC}	k_d[c] [10^5 s^{-1}]	ΔE_{ST}[d] [eV]
0.861	0.660	0.201	57	1.1	1.16	1.87	4.11	0.107	0.234	8.53	0.023

[a] Absolute PLQYs in toluene solution under nitrogen atmosphere (5×10^{-5} M) determined by an integrating sphere; [b] The prompt and delayed fluorescence lifetimes in toluene solution under nitrogen atmosphere (5×10^{-5} M) at room temperature; [c] The rate constants of the prompt fluorescence, IC or ISC processes, the delayed fluorescence; [d] Calculated energy gap of S_1 and T_1.

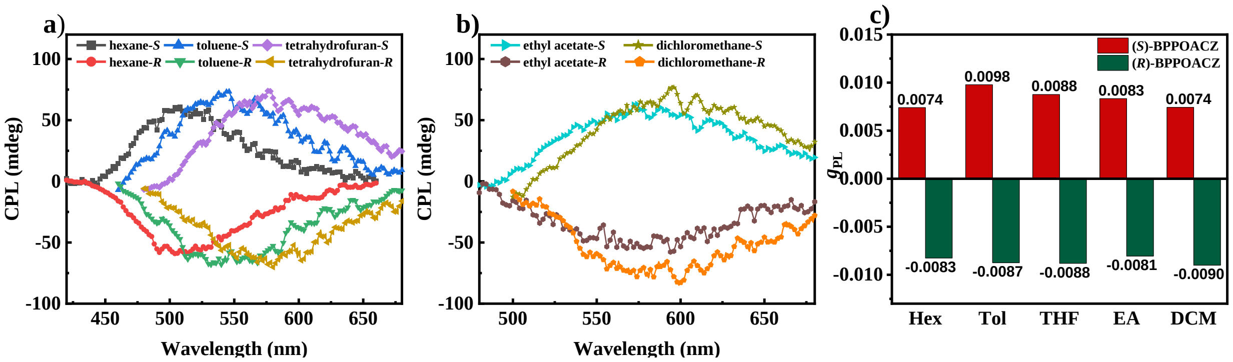

Figure S13. CPL spectra of (*R/S*)-BPPOACZ and g_{PL}s at the maximum emission wavelengths in different solutions.

10. Device fabrication and characterization

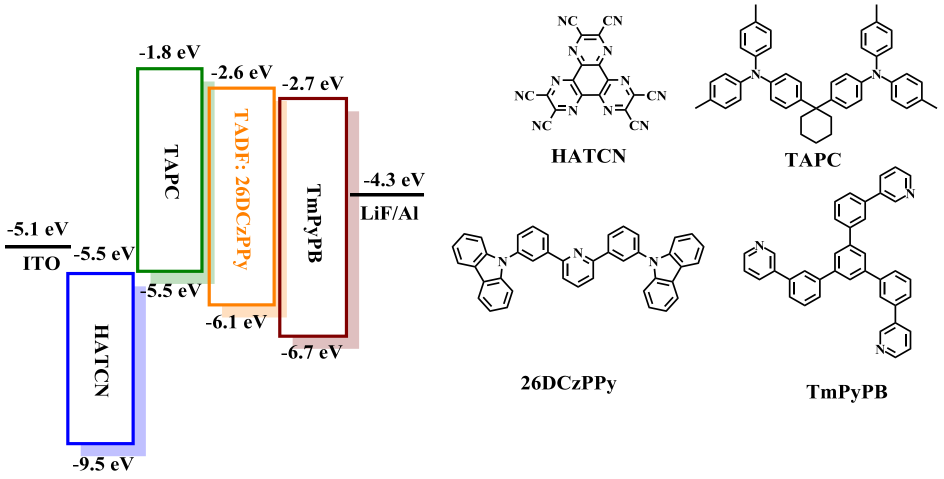

Figure S14. The energy diagram and the molecular structures of the compounds used in the device.

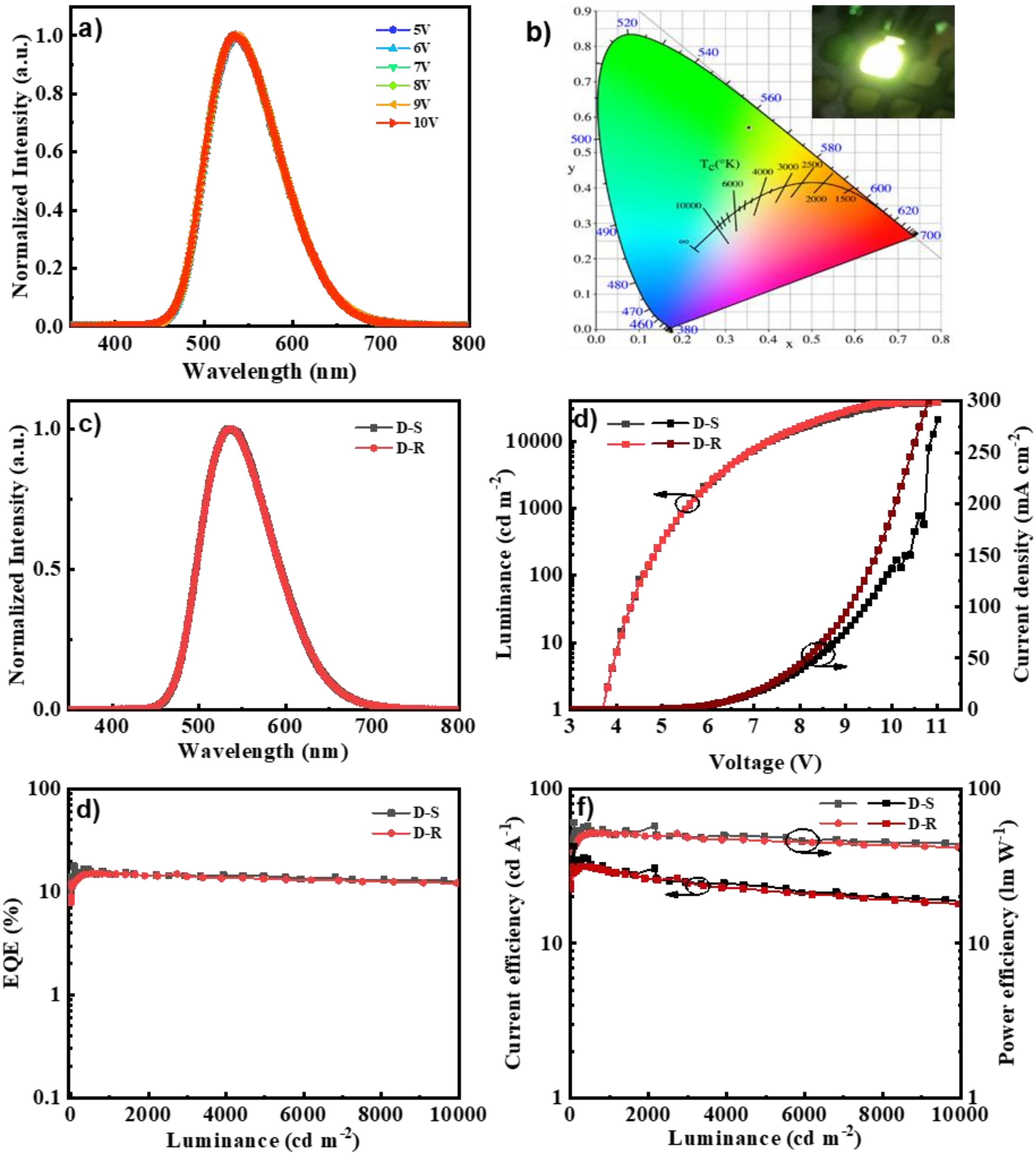

Figure S15. a) Electroluminescence spectra of device D-RAC at various voltages; b) Commission Internationale de L'Eclairage (CIE) 1931 coordinates of EL spectrum of device D-RAC based on (*rac*)-BPPOACZ and lighting by device; c) EL spectrum, d) current density - luminance - voltage curves, e) EQE-luminance curves and f) current-efficiency/ power-efficiency-luminance curves for devices D-S and D-R.

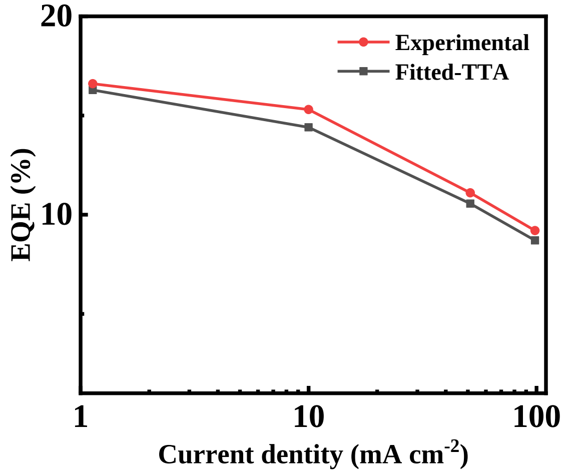

Figure S16. EQE-current density curve simulation for BPPOACZ device. The fitting results are based on triplet-triplet annihilation (TTA) models.

We investigated the efficiency roll-off behaviours by taking triplet-triplet annihilation (TTA) into account. The TTA model represents an exciton-exciton quenching process and the current density dependent EQE in the TTA model is expressed by equation (1):

$$\frac{\eta_{TT}}{\eta_0} = \frac{J_0}{4J}\left(\sqrt[2]{1+\frac{8J}{J_0}}-1\right)$$

where η_{TT} is the EQE in the presence of TTA, η_0 is the maximum EQE, J_0 is the current density where EQE reaches half of the maximum value.

References:

[1] Y. Wang, Y. Zhang, W. Hu, Y. Quan, Y. Li, Y. Cheng, *ACS Appl. Mater. Interfaces* **2019**, *11*, 26165 - 26173.

[2] S. Feuillastre, M. Pauton, L. Gao, A. Desmarchelier, A. J. Riives, D. Prim, D. Tondelier, B. Geffroy, G. Muller, G. Clavier, G. Pieters, *J. Am. Chem. Soc.* **2016**, *138*, 3990 - 3993.

[3] F. Song, Z. Xu, Q. Zhang, Z. Zhao, H. Zhang, W. Zhao, Z. Qiu, C. Qi, H. Zhang, H. H. Y. Sung, I. D. Williams, J. W. Y. Lam, Z. Zhao, A. Qin, D. Ma, B. Z. Tang, *Adv. Funct. Mater.* **2018**, *28*, 1800051.

[4] S. Sun, J. Wang, L. Chen, R. Chen, J. Jin, C. Chen, S. Chen, G. Xie, C. Zheng, W. Huang, *J. Mater. Chem. C* **2019**, *7*, 14511 - 14516.

[5] Y.-F. Wang, H.-Y. Lua, C. Chen, M. Li, C.-F. Chen, *Org. Electron.* **2019**, *70*, 71 - 77.

[6] N. Sharma, E. Spuling, C. M. Mattern, W. Li, O. Fuhr, Y. Tsuchiya, C. Adachi, S. Bräse, I. D. W. Samuel, Z.-C. Eli, *Chem. Sci.* **2019**, *10*, 6689 - 6696.

[7] M. Li, S.-H. Li, D. Zhang, M. Cai, L. Duan, M.-K. Fung, C.-F. Chen, *Angew. Chem. Int. Ed.*, **2018**, *57*, 2889 - 2893; *Angew. Chem.* **2018**, *130*, 2939 - 2943.

[8] Z.-G. Wu, H.-B. Han, Z.-P. Yan, X.-F. Luo, Y. Wang, Y.-X. Zheng, J.-L. Zuo, Y. Pan, *Adv. Mater.* **2019**, 1900524.

[9] C.-F. Chen, M. Li, Y.-F. Wang, D. Zhang, L. Duan, *Angew. Chem. Int. Ed.* **2020**, *59*, 3500 - 3504.
